# Comparative transcriptomic analysis reveals distinct responses of beneficial bacterial endophytes to wild and cultivated rice root exudates

**DOI:** 10.1007/s00299-026-03936-0

**Published:** 2026-08-20

**Authors:** Francesca Vaccaro, Maria Laura Amenta, Iacopo Passeri, Camilla Fagorzi, Stefano Varriale, Danuše Tarkowská, Aleš Pěnčík, Ivan Petřík, Federica Brunoni, Vittoria Brambilla, Andrea Rossoni, Erica Mica, Giampiero Valè, Elena Perrin, Alessio Mengoni, Roberto Defez, Carmelina Bianco

**Affiliations:** 1 Department of Biology, University of Florence, Sesto Fiorentino, Italy; 2 Institute of Bioscience and BioResources National Research Council of Italy, Naples, Italy; 3 Laboratory of Growth Regulators, Faculty of Science, Palacký University Olomouc, Šlechtitelů 27, 77900 Olomouc, Czech Republic; 4 Laboratory of Growth Regulators, Institute of Experimental Botany of the Czech Academy of Sciences, Šlechtitelů 27, 77900 Olomouc, Czech Republic; 5 Department of Agricultural and Environmental Sciences, University of Milan, Via Celoria 2, 20133 Milan, Italy; 6 Department for Sustainable Development and Ecological Transition, Università del Piemonte Orientale, Piazza Sant’Eusebio, 5, 13100 Vercelli, Italy; 7 Department of Biotechnology, University of Verona, Strada le Grazie, 15, 37134 Verona, Italy

**Keywords:** Bioinoculants, *Oryza sativa*, *Oryza rufipogon*, Transcriptome, Hormonomics, Plant growth-promoting bacteria

## Abstract

**Key message:**

Root exudates from Oryza rufi pogon elicit stronger transcriptional responses in benefi cial bacterial endophytes and, together with bacterial inoculation, reveal distinct plant responses compared with cultivated rice, suggesting that microbiome-associated traits altered during domestication could be exploited for sustainable rice breeding.

**Abstract:**

Beneficial interactions between plants and microorganisms strongly influence plant health and productivity, and root exudates play a central role in shaping these associations. In this study, we analyzed the transcriptional responses of the bacterial endophytes *Enterobacter asburiae* RCA24 and *Kosakonia sacchari* RCA25 to root exudates from two commercial Italian rice accessions (*Oryza sativa* Baldo and Vialone Nano) and from an accession of the wild progenitor of tropical rice, *Oryza rufipogon*. Transcriptome analysis showed that RCA24 displayed distinct responses to the two *O. sativa* varieties, whereas RCA25 exhibited more extensive transcriptional changes in response to *O. rufipogon* root exudates. Differentially expressed genes were mainly associated with central metabolism, stress response, and signal transduction, suggesting distinct patterns of bacterial adaptation to the different exudate profiles. Transcriptome analysis of inoculated rice further indicated broader transcriptional changes in plants colonized by RCA24 than in those colonized by RCA25. Differentially expressed genes, particularly in shoots, were associated with defense responses, hormone-mediated signaling pathways, and ribosome biogenesis, consistent with genotype-dependent plant responses to different bacterial strains. Overall, these findings indicate that wild and cultivated rice accessions differ in their interaction with beneficial bacterial endophytes at the transcriptional level. Traits associated with plant–microbiota interactions in *O. rufipogon*, which are lost during domestication and diversification, may represent valuable targets for future studies aimed at enhancing beneficial microbial associations in cultivated rice.

**Supplementary Information:**

The online version contains supplementary material available at https://doi.org/10.1007/s00299-026-03936-0.

## Introduction

Plant–microbe interactions are fundamental for plant health and productivity (Hassani et al. 2018). Crop yield and resilience are influenced by multiple factors, including plant genotype, agronomic practices, the environment, and the crop-associated microbiome (Berg 2009; Vandenkoornhuyse et al. 2015). Identifying plant genotypes that are more tolerant to abiotic and biotic stresses, highly productive, and capable of further benefiting from positive interactions with microorganisms is central to what is known as microbiome-driven or microbiome-assisted plant breeding (Nerva et al. 2022). Microorganisms can provide plants with molecules necessary for growth, which are often supplied externally through fertilization, and can protect against pathogens’ attack. Efficient plant–microbe symbioses rely on complex interactions between the genotypes of both partners, which remain poorly understood. Rice is the most widely consumed crop by humans worldwide and is harvested on approximately 160–170 million ha (FAOSTAT, 2024), second only to maize. Italy is the leading rice producer in Europe, with 226,000 ha cultivated in 2024 (Ente Nazionale Risi), representing 50–55% of the total rice cultivated area in Europe (FAOSTAT, 2024). Cultivated rice belongs to the species *O. sativa*, which was domesticated from its wild progenitor, *O. rufipogon*, a species endemic to the wetlands of tropical and subtropical Asia (Gutaker et al. 2020). Italian rice cultivars, including the commercial varieties used in this study, ‘Baldo’ and ‘Vialone nano’, generally belong to the temperate *japonica* group, which diverged from *indica* following domestication and subsequently evolved along independent trajectories. *O. rufipogon* harbors greater allelic diversity than domesticated *O. sativa* and serves as a source of much of the genetic variation lost during rice domestication and breeding (Izawa 2022).

Domestication has influenced the composition of the root-associated microbiome (Escudero-Martinez and Bulgarelli 2019; Pérez-Jaramillo et al. 2018; Raaijmakers and Kiers 2022). Although genotypic differences in rice have been shown to significantly affect root-associated microbial communities (Edwards et al. 2015; Xiong et al. 2021; Xu et al. 2020), rice breeding programs have so far paid limited attention to the root-associated microbiome. Root exudates mediate the first contact between roots and the soil microbiota (Korenblum et al. 2020). They comprise a diverse array of molecules secreted by plants into the rhizosphere, the soil zone immediately surrounding the roots. These exudates can serve both as a nutrient source for microorganisms and as signaling molecules that influence microbial behavior and gene expression (Korenblum et al. 2020; Zhalnina et al. 2018; Poole et al. 2018). Different host plant genotypes produce root exudates with distinct chemical compositions, leading to distinct symbiotic interactions (Kiers et al. 2007; Pacheco-Moreno et al. 2024; Weese et al. 2015).

By studying the transcriptomic response of the rhizobium *Sinorhizobium meliloti* to root exudates from different *Medicago sativa* (alfalfa) cultivars, Fagorzi et al. (2021) demonstrated that nearly 30% of the total differentially expressed genes could be statistically associated with interactions between specific plant and bacterial genotypes (Fagorzi et al. 2021). Similar evidence has recently been reported for the transcriptomic response in interactions between bacteria and fungi belonging to the plant microbiota (Vaccaro et al. 2024), suggesting that specificity of genotype-by-genotype interactions is a key determinant of the successful plant microbiota assembly. We speculate that a parallel mechanism may also occur in endophyte-rice interactions. Whole transcriptome analysis aimed at uncovering the molecular networks underlying rice symbiotic relationships has highlighted several key players, including genes involved in secondary metabolism, immunity, and phytohormones (King et al. 2023), as well as ammonium and sugar transport (Thomas et al. 2019).

To investigate the molecular basis underlying the differential endophytic colonization ability observed among rice genotypes, we analyzed the early transcriptomic response associated with the perception of root exudates. To test this hypothesis, we employed an experimental model comprising two endophytic plant growth-promoting bacterial strains (*E. asburiae* RCA24 and *K. sacchari* RCA25), together with three rice genotypes: the wild accession *O. rufipogon* 602-131-2 (tropical rice) and the *O. sativa* cultivars ‘Vialone Nano’ and ‘Baldo’, two commercially important temperate Italian rice varieties. These bacterial strains were previously shown to exhibit different colonization efficiencies across the three rice genotypes (Andreozzi et al. 2019).

The results showed that both bacterial strains underwent transcriptomic reprogramming in response to exudates from specific rice genotypes, differing both in the extent of the response (0.1–20% of expressed genes differently regulated) and in the functional categories affected. The exudates from the wild *O. rufipogon* accession induced changes in a larger number of signal transduction-related genes than those from the cultivated varieties. These differences should be interpreted as variations in transcriptional responsiveness rather than evidence of enhanced functional outcomes. We also verified that the three rice genotypes released distinct phytohormone profiles in the rhizosphere, with greater genotypic differentiation for gibberellins than for auxins. Overall, our results support the existence of a genotype-dependent molecular communication between rice plants and endophytic bacteria, characterized by reciprocal transcriptional responses and distinct signaling signature during early stage of interaction.

## Materials and methods

### Bacterial strains and culture conditions

*E. asburiae* RCA24 and *K. sacchari* RCA25 were originally isolated from *O. sativa* temperate *japonica* cv. Volano rice plants grown in Italy (Defez et al. 2017). The strain RCA24 and RCA25 previously reported as *Herbaspirillum huttiense* and *Enterobacter cloacae* (Andreozzi et al. 2019)*,* have since been reclassified as *E. asburiae and K. sacchari,* respectively. *E. asburiae* RCA24 was shown to produce indole-3-acetic acid, while *K. sacchari* RCA25 harbors nitrogen-fixation genes and fixes atmospheric nitrogen inside rice plants (Bianco et al. 2021). Strains were grown at 30 °C in LB medium containing 20 µg ml^−1^ vancomycin. Thereafter, the two strains are indicated as RCA24 and RCA25 only.

Genome sequences and annotations of RCA24 and RCA25 strains can be accessed from Bioproject PRJNA1120238 (assemblies ASM4012233v1 and ASM4012231v1, respectively).

### Plant materials

Three rice accessions were examined in this study: *O. sativa* ‘Vialone Nano’ and *O. sativa* ‘Baldo’, which are commercial varieties of Italian rice within the temperate *Japonica* group, and *O. rufipogon* accession 602-131-2, (kindly provided by IRRI, International Rice Research Institute, Metro Manila, Philippines).

### Rice colonization efficiency

Rice seeds were surface sterilized by incubation in 70% ethanol for 8 min, followed by 5% sodium hypochlorite solution containing 0.1% Tween 20 for 25 min. Seeds were then washed several times with sterilized distilled water and incubated for 5 days at 21 °C in the dark for germination. Germinated seeds were transferred into plastic pots containing inorganic substrates (sand with a 1.2–0.8 mm granule size and perlite with a 3–4 mm granule size) in a 1:1 ratio. Each pot was kept in the growth chamber under long-day conditions (16 h), at 19–23 °C, and 75% relative humidity. 21 days after inoculation (DAI), plants were carefully uprooted from pots, washed by dipping them in tap water, and weighed. Surface sterilization of whole plants and tissue homogenization were carried out as described in Bianco et al. (2021). Dilutions of the homogenates (1:10,000) were spread onto 1.5% LB agar plates containing the specific antibiotic (20 μg mL^−1^ vancomycin) and incubated at 30 °C. After 24 h, CFU were counted.

### Seeds inoculation and plant growth

Dehulled seeds of rice plants (‘Baldo’, ‘V. Nano’, and ‘Rufipogon’) were surface-sterilized and germinated as described by Amenta et al. (2024). After 5 days, the germinated seeds were incubated in Petri dishes containing 50 mL of 1 × PBS solution supplemented with each strain to a final concentration of 10^7^ cells mL^−1^ for 4 h at room temperature. Seeds incubated in 1 × PBS alone were used as a control. The inoculated seeds were then transferred into plastic pots containing a 1:1 mixture of sand (0.8–1.2 mm granule size) and perlite (3–4 mm granule size) as a substrate. The pots were maintained in a growth chamber under long-day (16 h) conditions, at 19° C and 75% relative humidity, and watered daily. Once a week, plants received a nitrogen-free nutrient solution (Bianco et al. 2021). Six replicates were established for each rice ecotype.

### Root exudates’ preparation and bacterial treatment

For exudate collection, the roots of non-inoculated, intact 3-week-old plants were carefully removed from pots, washed throughout to remove any remaining soil, transferred to 30 mL Milli-Q water, and incubated for 48 h under greenhouse conditions. After incubation, the root exudate solutions were filtered through a 0.22 μm Millipore filter membrane to remove any root debris, freeze-dried, and stored at −80 °C until hormonal analysis. Aliquots of non-freeze-dried samples were used to treat RCA24 and RCA25 bacterial cultures.

Overnight cultures of both strains were diluted with fresh LB medium. When the OD_600_ reached 0.6, 0.5 ml of root exudates collected from rice plants was added. An equal amount of Mili-Q water was used as the control treatment. The volume of root exudates was selected, because it did not produce any detectable effect on bacterial growth. Cultures were grown at 30 °C with shaking at 200 rpm for 2.5 h, after which cells were harvested for RNA isolation. For each strain and treatment, three biological replicates were prepared, resulting in a total of 12 samples per strain.

### Detection and quantification of phytohormones in root exudates

Sample preparation and analysis of gibberellins (GAs) were performed according to the method described in Urbanová et al. (2013) with some modifications. Briefly, rice root exudate samples evaporated to dryness were dissolved in 1 mL of ice-cold 80% acetonitrile containing 5% formic acid as the extraction solution and then sonicated for 5 min in a laboratory ultrasonic bath. The samples were then extracted overnight at 4 °C using a benchtop laboratory rotator Stuart SB3 (Bibby Scientific Ltd., UK) after adding 2 pmol of [^2^H_2_]GA_1_, [^2^H_2_]GA_4_, [^2^H_2_]GA_9_, [^2^H_2_]GA_19_, [^2^H_2_]GA_20_, [^2^H_2_]GA_24_, [^2^H_2_]GA_29_, [^2^H_2_]GA_34_, and [^2^H_2_]GA_44_ (OlChemIm, Czech Republic) as internal standards. The homogenates were centrifuged at 36,670 *g* and 4 °C for 10 min (Hermle Z 35 HK, Hermle Labortechnik GmbH, Germany), and corresponding supernatants were further purified using mixed-mode SPE cartridges (Waters, Ireland). Following SPE purification, samples were evaporated to dryness under a stream of nitrogen (Turbovap^®^ LV, Biotage, Sweden), redissolved in the initial mobile phase, and analyzed by ultra-high-performance liquid chromatography–tandem mass spectrometry (UHPLC–MS/MS; Waters, USA) using an Acquity UPLC I-Class Plus system (Waters, USA) coupled to a triple quadrupole mass spectrometer Xevo TQ-XS (Waters, USA). GAs were detected in multiple-reaction monitoring mode by monitoring the transition of the [M–H]- ion to the appropriate product ion, as described in Urbanová et al. (2013). Data were processed using Masslynx 4.2 software (Waters, USA). GA concentrations were determined using the standard isotope dilution method (Rittenberg and Foster 1940).

Auxin metabolites were quantified as described by Pěnčík et al. (2018). Dried root exudate samples were dissolved in 1 mL 50 mmol/L phosphate buffer (pH 7.0) containing 0.1% sodium diethyldithiocarbamate and stable isotope-labeled internal standards. Aliquots (200 µL) were either acidified to pH 2.7 and purified by in-tip µSPE or derivatized with cysteamine prior to acidification and µSPE for indole-3-pyruvic acid (IPyA) determination. The eluates were evaporated to dryness, reconstituted in 10% aqueous methanol, and analyzed using an HPLC system 1290 Infinity III (Agilent Technologies, USA) equipped with Kinetex C18 column (50 mm × 2.1 mm, 1.7 µm; Phenomenex) and linked to 6495D Triple Quad detector (Agilent Technologies, USA).

Phytohormone concentrations were log₁₀(x + 1)-transformed prior to multivariate analysis to reduce the influence of extreme values and homogenize variance. Variables showing zero variance across samples were excluded from the analysis. Principal Component Analysis (PCA) was performed on mean-centered, non-scaled data using the *prcomp* function in R, and results were visualized as biplots using the *ggplot2* and *ggfortify* packages. Differences in phytohormone profiles among genotypes were assessed by permutational multivariate analysis of variance (PERMANOVA) using the *adonis2* function from the *vegan* package, with genotype as the explanatory variable and 999 permutations. Mean concentrations ± standard deviations were calculated for each compound and genotype from three biological replicates. All analyses were performed in R (v.4.4.2).

### RNA preparation for RNA-seq analysis

Bacterial cultures of RCA24 and RCA25 were grown in LB medium to the exponential phase (OD_600_ = 0.7) and then treated with root exudates from *O. sativa* ‘Vialone Nano’, *O. sativa* ‘Baldo’, and *O. rufipogon.* Untreated cultures were used as controls. RNA was extracted from RCA24 and RCA25 using the PureLink RNA Mini Kit (Invitrogen) according to the manufacturer’s instructions, with the following modification: proteinase K was added to the lysis buffer at a final concentration of 75 µg ml^−1^. Residual genomic DNA was removed using the RNase-free TURBO DNase kit (Ambion) according to the manufacturer’s instructions. RNA quality was assessed by measuring the RNA Integrity Number (R.I.N.) using a TapeStation automated electrophoresis apparatus (Agilent, CA, USA).

For plant RNA preparation, roots and leaves were collected from 2-week-old non-inoculated plants from those inoculated with RCA24- and RCA25, immediately frozen in liquid nitrogen, and stored at −80 °C until use. Total RNA was extracted from frozen material (100 mg) using the Trizol Reagent (Invitrogen) according to the manufacturer’s instructions. Residual genomic DNA was removed using the RNase-free TURBO DNase kit (Ambion) according to the manufacturer’s instructions. RNA quality was assessed by measuring the R.I.N. using an Agilent 2100 Bioanalyzer and the Caliper GX system.

### RNA sequencing and analysis

A bacterial RNA sequencing library was constructed after rRNA depletion using the RiboZero kit (Illumina) with the TruSeq Stranded Total RNA kit (Illumina). Sequencing was performed by IGA Technology Service (Udine, Italy). Plant RNA sequencing library preparation and sequencing was performed by BMKGENE technology service (Münster, Germany).

Raw bacterial RNA-seq reads were subjected to quality control and preprocessed using fastp (v0.23.4) to ensure high-quality data for downstream analysis. fastp was used with default settings. The quality of reads before and after preprocessing was assessed using FastQC (v0.12.1). A reference-guided transcripts assembly was then performed. All reads datasets were aligned to their respective indexed reference genomes using HISAT2 (v2.2.1) with default parameters in reference-guided mode (Kim et al. 2019). Sequence alignment map files (SAM) were subsequently converted to BAM format, sorted, indexed, and merged using SAMtools (Li et al. 2009). The assemblies were run using StringTie2 (v2.2.3) (Kovaka et al. 2019) with the “-G” option to include the annotation in the merging (refer to “Annotation” paragraph for more details) and to obtain the Gene Transfer Format (GTF) of covered transcripts. Because the reference annotation was supplied through the “-G” option, all annotated loci were retained in the merged set irrespective of their expression level, so that lowly expressed genes were preserved and quantified rather than lost during assembly; the procedure was therefore reference-guided and not de novo (Pertea et al. 2016). The transcriptome in FASTA format was then extracted by cross-referencing the GTF and the reference genome using gffread (v0.12.8) (Pertea and Pertea 2020). The genome annotations were performed using Prokka (1.14.6) with default options (Seemann 2014). Salmon (v1.10.1) (Patro et al. 2017) was used to estimate the transcripts abundances. After the transcriptome indexing, quantification was obtained by running Salmon with the “– validateMappings” option for selective alignment. With selective alignment, Salmon does not discard multi-mapping reads but assigns them probabilistically through a bias-aware expectation–maximization model that also corrects for sequence-specific, positional and fragment-GC biases (Patro et al. 2017; Srivastava et al. 2020). As the quantification targets were coding sequences extracted from the reference genomes, the resulting counts correspond to the annotated gene complement of each strain (4309 and 4575 genes for RCA24 and RCA25, respectively) and are therefore unaffected by redundant or duplicated assembled sequences. Differential abundance analysis was performed using DESeq2 (v1.36.0) package (Love et al. 2014). Differentially expressed genes (DEGs, |log2FC|≥ 1, adjusted *p* value < 0.05) of RCA24 and RCA25 were further functionally annotated using the online version of eggNOG-mapper version 2 with default settings (Cantalapiedra et al. 2021), which assigns KEGG Orthology (KO) identifiers and Clusters of Orthologous Groups (COG) based on sequence homology against the eggNOG database. For each pairwise contrast, the number of DEGs mapping to each KEGG pathway was counted using the KEGG_Pathway field from the eggNOG-mapper output, retaining only entries with a valid ‘map’-prefixed pathway identifier. Pathway names were retrieved programmatically via the KEGGREST R package (Tenenbaum 2025). Similarly, the percentage of up- and downregulated DEGs assigned to each COG category was calculated relative to the total number of annotated DEGs in the corresponding direction. Bar plots were generated using the ggplot2 package (Wickham 2009). To complement this threshold-based analysis, a cutoff-free gene-set enrichment analysis (GSEA) was performed for each contrast and strain with the fgsea R package (v[FGSEA VERSION]) (Korotkevich et al. 2021). All tested genes were ranked by the DESeq2 Wald statistic, without applying any significance or fold-change filter, and tested against gene sets built from the eggNOG-mapper KEGG pathway and COG category assignments (gene-set size restricted to [minSize]-[maxSize] genes). *P* values were adjusted with the Benjamini–Hochberg procedure and gene sets with an adjusted *p* value < 0.05 were considered significantly enriched (Table S6). KEGG global and overview maps (map01100, map01110, map01120, map01130) were retained in the output but are not interpreted as individual pathways. In addition, the sensitivity of DEG detection to the fold-change threshold was assessed by re-extracting DEGs for all contrasts under three schemes, all with an adjusted *p* value < 0.05: |log2FC|≥ 1 (as reported here), a relaxed |log2FC|≥ 0.5, and no fold-change cutoff (Table S7). Functional enrichment was obtained using eggNOG mapper across the entire gene set, annotating for GOs covering molecular functions, KEGG pathways, biological processes, and cellular components. To retrieve the aminoacidic sequences required by eggNOG mapper, the Seqtk (v1.4) tool has been used with a list of the collected coding sequences (CDS). All statistical analyses were performed in R Studio (v4.4.2) (RStudio Team, 2020). Principal Component Analysis (PCA) of bacterial transcriptomes was performed on variance-stabilizing transformed (VST) expression values using the plotPCA function of the DESeq2 package, prior to differential expression analysis and DEG selection, based on the top 500 most variable genes across all samples (default parameters); results were visualized using the ggplot2 package (Wickham 2009). Venn diagrams were obtained with *ggvenn* package.

Raw plant RNA-seq reads were preprocessed and cleaned by removal of adaptor sequences, low-quality-end trimming, and low-quality reads using fastp (v0.23.4) with default settings. Transcript-level quantification was performed with Salmon (v1.10.1) (Patro et al. 2017) against the CDS reference transcriptome deposited on Ensembl Plants to restrict quantification to protein-coding regions. The quantification results were collected with custom scripts. The resulting gene–counts matrix was imported into DESeq2 (v1.36.0) package (Love et al. 2014) for downstream analysis. Genes were considered significantly differentially expressed if they met the thresholds of an adjusted *p* value < 0.05 and |log2FC|≥ 1. Data analysis and visualization were performed in R (v4.4.2). Gene annotations were retrieved from Ensembl Plants and intersected with the set of DEGs for functional characterization. To identify significantly overrepresented functional categories within DEGs, enrichment analysis was performed using COG categories via the eggNOG-mapper web server (version 2) with default parameters. COG categories provided a compact, non-redundant categorization scheme that facilitated visualization of general enriched functions within DEGs. Nested likelihood ratio tests (LRTs) were performed as indicated previously (Fagorzi et al. 2021). The DEGs identified as significant in the interaction model were used to perform Gene Ontology (GO) enrichment analysis with *topGO* (Alexa and Rahnenfuhrer 2024) to provide a pathway-centered analysis for enrichment relative to those DEGs.

## Results

### *E. asburiae* RCA24 and *K. sacchari* RCA25 differently colonized rice plants

Quantitative evaluation of endophytic colonization revealed sharp differences in host preference. RCA25 efficiently colonized *O. rufipogon* and ‘Baldo’ (12.9-fold and 3.2-fold more than RCA24, respectively), while RCA24 showed a tenfold preference for ‘Vialone Nano’ (Table 1).

**Table 1:** Colonization efficiency of rice plant genotypes by RCA24 and RCA25

Rice variety	*E. asburiae* RCA24 CFU/g^a^	*K. sacchari* RCA25 CFU/g^a^	*Ratio* *RCA25 vs. RCA24*
*O. sativa* Baldo	6.8 ± 1.3 x${10}^{8}$	22.0 ± 3.7 x${10}^{8}$	3.2 ± 0.8
*O. sativa* Vialone Nano	24.5 ± 4.1 x${10}^{8}$	2.4 ± 0.6 x${10}^{8}$	0.10 ± 0.03
*O. rufipogon*	9.5 ± 1.0 x${10}^{8}$	123 ± 15 x${10}^{8}$	12.9 ± 2.1

^**a**^Mean and standard deviation of colonization efficiency (CFU/g fresh weight) for six independent biological replicates

### Differentiated transcriptional response of *E. asburiae *RCA24 and *K. sacchari* RCA25 to rice root exudates

**Fig. 1:**
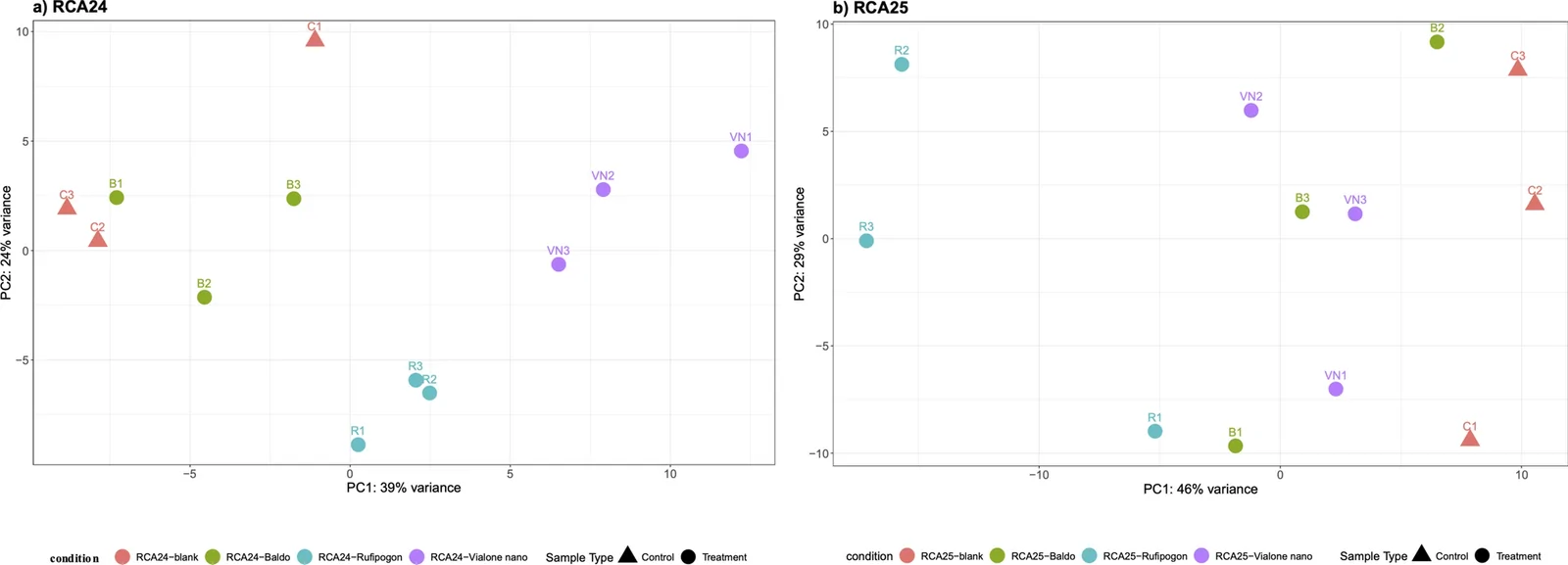
Principal Component Analysis (PCA) of VST-normalized expression values, performed prior to differential expression analysis and DEG selection. Bacterial strains RCA24 (**a**) and RCA25 (**b**) were exposed to root exudates of *O. sativa* genotypes ‘Baldo’ (B) and ‘Vialone Nano’ (VN), and *O. rufipogon* (R), compared with the control condition (C). Each point represents one biological replicate (*n* = 3 per treatment), and centroids indicate the mean position of replicates within each treatment group. Percentages on the axes indicate the variance explained by each principal component. *B* Baldo, *R* Rufipogon, *VN* Vialone Nano, *C* control

**Fig. 2:**
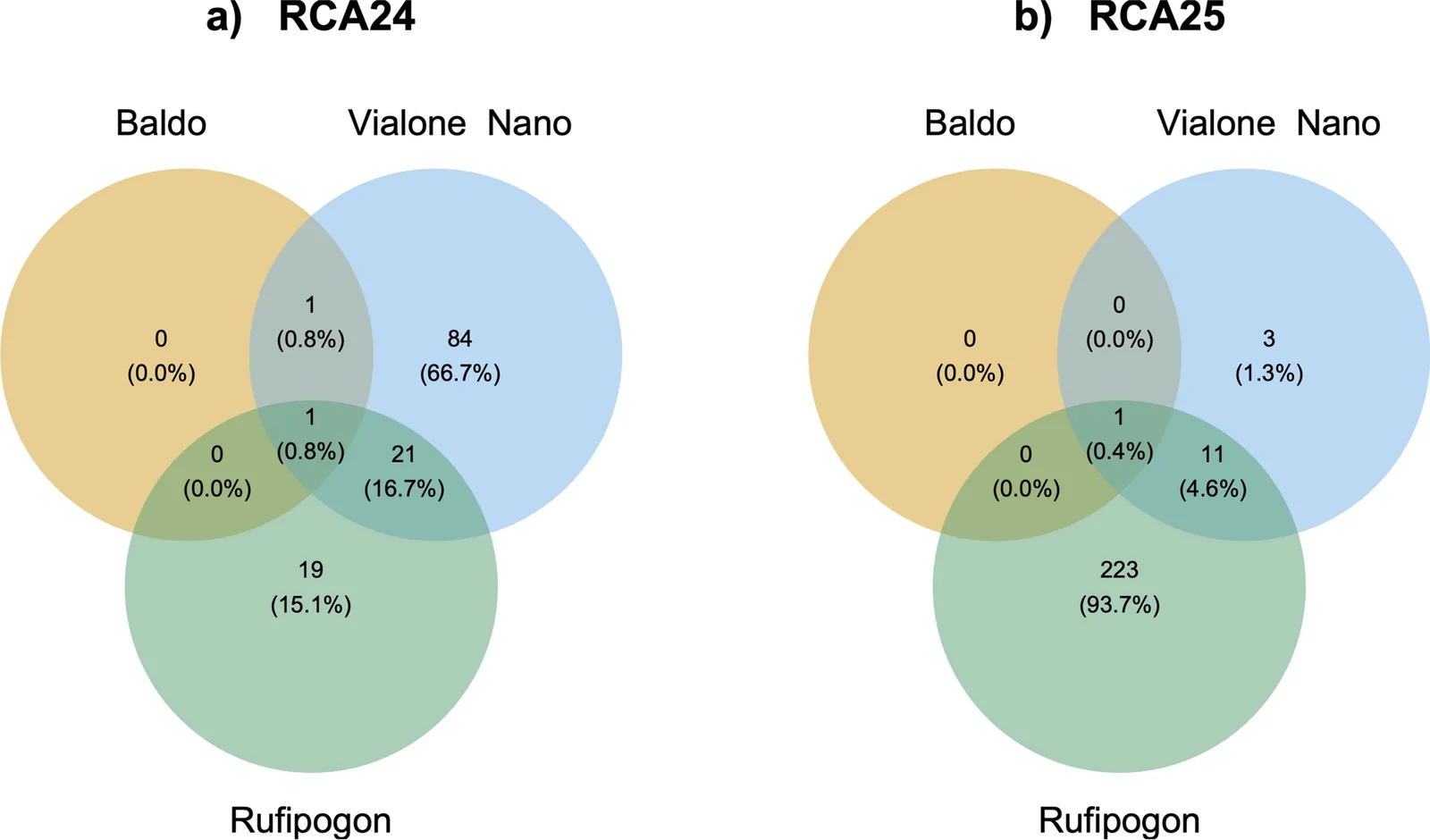
Venn diagrams showing unique and shared DEGs identified in RCA24 and RCA25 in response to rice root exudates relative to the control condition. DEGs were defined as genes with |log2FC|≥ 1 and adjusted *p* value (FDR) < 0.05. Percentages are calculated relative to the total number of DEGs identified for RCA24 (**a**) and RCA25 (**b**). Differential expression analysis was performed using DESeq2, with *n* = 3 biological replicates per condition

**Fig. 3:**
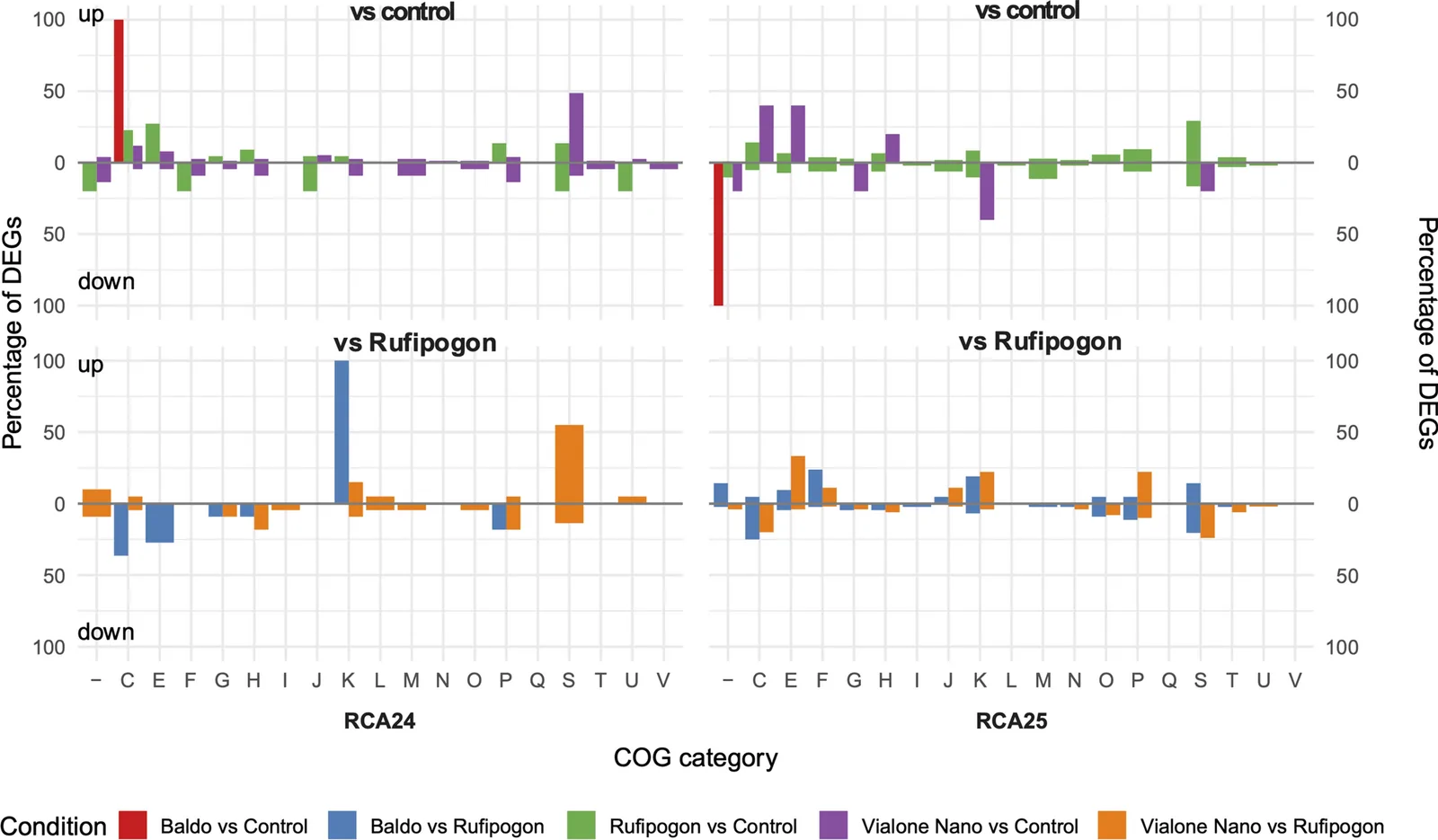
Distribution of upregulated and downregulated DEGs across COG functional categories in RCA24 (left) and RCA25 (right). Percentages refer to the proportion of DEGs assigned to each COG category relative to the total number of annotated DEGs in the corresponding comparison. DEGs were identified using DESeq2 (v1.36.0) package, applying a threshold of |log2FC|≥ 1 and FDR < 0.05. Comparisons were performed against the control condition and against the *O. rufipogon* treatment. COG category descriptions are reported in Table S1

Transcriptome analysis of RCA24 and RCA25 exposed to root exudates revealed extensive transcriptional reprogramming. Analysis of differentially expressed genes (DEGs) (Dataset S1) indicated that *O. rufipogon* exudates elicited nearly 6 times more DEGs in RCA25 than in RCA24 (Table 2). PCA of VST-normalized expression values, performed prior to differential expression analysis and independently of DEG selection, indicated that bacterial transcriptional responses were strongly influenced by plant genotype (Fig. 1). Comparison of the transcriptomic responses of RCA24 and RCA25 (Fig. 2) revealed that treatment with ‘Vialone Nano’ root exudates accounted for 66.7% of all identified DEGs in RCA24, whereas treatment with *O. rufipogon* root exudates induced the highest number of DEGs in RCA25, representing 93.7% of the total. In contrast, treatment with ‘Vialone Nano’ exudates resulted in the fewest unique DEGs in RCA25, accounting for only 1.3% of the total DEGs. The functional composition of the DEG datasets varied according to both bacterial strain (RCA24 or RCA25) and comparison (*O. sativa* and *O. rufipogon* vs control or *O. sativa* vs *O. rufipogon* root exudates) (Fig. 3, Table S1). The COG category S (Function unknown) was highly represented in all datasets, indicating that many gene potentially involved in bacteria–plant interactions remain poorly characterized. In general, categories related to central metabolism ([E] amino acid transport and metabolism; [C] energy production and conversion; [G] carbohydrate transport and metabolism; [H] coenzyme transport and metabolism) were detected in most comparisons among both up- and downregulated DEGs (Dataset S1). Genes associated with two-component system of signal transduction system were identified in nearly all comparisons involving *O. rufipogon* exudates, whereas this pathway was identified only for RCA24 treated with ‘Vialone Nano’ exudates vs control (Dataset S2). These results suggest that *O. rufipogon* induced a more extensive remodeling of bacterial cellular functions than exudates from *O. sativa* varieties. Nitrogen metabolism was downregulated in RCA25 in both ‘Baldo’ and ‘Vialone Nano’ vs *O. rufipogon* comparisons, whereas it was upregulated in RCA24 exposed to ‘Vialone Nano’ and *O. rufipogon* exudates vs control. Cysteine and methionine metabolism was detected in RCA24 across multiple contrasts but not in RCA25 under the applied |log2FC|≥ 1 threshold; a cutoff-free analysis (see below) indicates that this pathway is modulated in RCA25 as well, at a magnitude insufficient for individual genes to pass the threshold (Table S7). At the individual gene level, iron transport gene *feoA* was upregulated in both RCA24 and RCA25 in response to *O. rufipogon* exudates vs control, representing one of the few convergent responses shared by the two strains. In contrast, the hydrogenase assembly gene *hypA* showed strain-specific regulation, being downregulated in RCA24 exposed to ‘Baldo’ exudates and upregulated in RCA25 exposed to *O. rufipogon* exudates. The purine biosynthesis gene *purK* was differently regulated in multiple contrasts in both strains. It was downregulated in RCA24 exposed to ‘Vialone Nano’ exudates and in RCA25 exposed to *O. rufipogon* exudates, whereas it was upregulated in RCA25 treated with ‘Baldo’ exudates. Opposite trend in amino acid metabolism was also observed in response to wild rice exudates: the aromatic amino acid biosynthesis gene *aroG* was downregulated in RCA25 exposed to *O. rufipogon* exudates compared to the control, whereas the methionine biosynthesis gene *metB* was upregulated in RCA24 under the same conditions. Among genes showing strain-specific regulation, the putative selenoprotein gene *ydfZ* and the peptidase gene *yhbU* were upregulated in RCA24 exposed to ‘Vialone Nano’ exudates vs control but downregulated in RCA25 in comparisons involving *O. rufipogon*. The nickel/cobalt stress response gene *yfgG* was consistently upregulated in RCA24 in comparisons involving ‘Vialone Nano’ exudates. To verify that these patterns were not an artifact of the |log2FC|≥ 1 threshold, DEGs were re-extracted for all contrasts under a relaxed (|log2FC|≥ 0.5) and a cutoff-free criterion (Table S7). The ranking of contrasts by number of DEGs was fully preserved for both strains at every threshold, with ‘Vialone Nano’ vs control the strongest contrast in RCA24 and *O. rufipogon* vs control the strongest in RCA25, and ‘Baldo’ vs control consistently the weakest. A complementary cutoff-free GSEA based on the DESeq2 Wald statistic detected significantly enriched KEGG pathways and COG categories in all contrasts, including ‘Baldo’ vs control (Table S6), indicating that coordinated, low-magnitude transcriptional shifts are present even where few individual genes reach significance. In the DEG-poor contrasts, however, the enriched sets were predominantly broad and generic (KEGG global and overview maps, high-level COG categories, ribosome, oxidative phosphorylation, and amino acid biosynthesis), a pattern compatible with a modest shift in overall physiological state rather than with a targeted, exudate-specific response. The functional interpretation presented below is therefore based on the threshold-based DEG sets.

**Table 2:** Number of DEGs identified in RCA24 and RCA25

Contrast	*E. asburiae* RCA24	*K. sacchari* RCA25
Baldo vs. control	2 (0.05%)	1 (0.02%)
Vialone Nano vs. control	107 (2.48%)	15 (0.32%)
*O. rufipogon* vs. control	41 (0.95%)	235 (5.13%)
Baldo vs *O. rufipogon*	15 (0.35%)	70 (1.53%)
Vialone Nano vs *O. rufipogon*	57 (1.32%)	63 (1.37%)

The percentages in relation to the total number of genes are reported in brackets (genes are 4309 and 4575 for RCA24 and RCA25, respectively)

### Phytohormones released into the rhizosphere by three rice genotypes exhibited distinct profiles

**Fig. 4:**
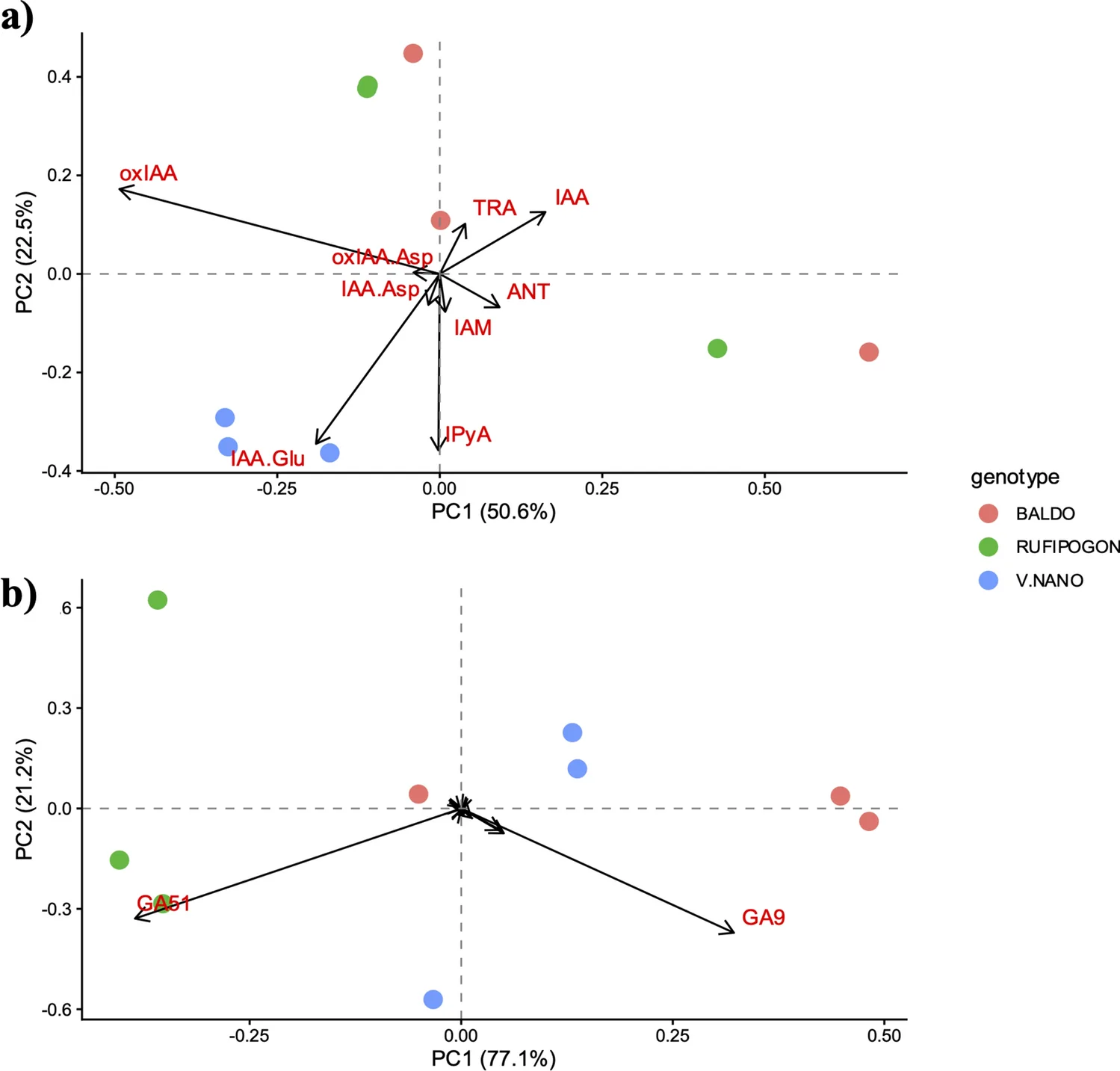
PCA biplots of auxin (**A**) and gibberellins (**B**) profiles in root exudates of the three rice genotypes. Each dot represents one biological replicate (*n* = 1 per genotype), and vectors indicate metabolite loadings contributing to sample separation. The percentages of variance explained by PC1 and PC2 are shown on the axes. PCA was performed using scaled metabolite abundance data

Root exudates and root extracts from *O. sativa* (‘Baldo’ and ‘Vialone Nano’) and *O. rufipogon* were analyzed separately for the presence and abundance (pmol/l) of auxins and gibberellins, two phytohormone classes frequently manipulated by plant-associated microorganisms to facilitate colonization and promote growth (Mukherjee et al. 2022). Hormones concentrations measured in roots exudates and root extracts are reported in Tables S2–S4. PCA analyses were performed separately for the two matrices (Fig. 4 and Fig. S1, respectively). In roots exudates, GA and auxin profiles differed among genotypes (Fig. 4A, B). For auxins, including both active compounds and inactive conjugated forms, PC1 and PC2 explained 50.6% and 22.5% of the total variance, respectively (Fig. 4A). Similarly, genotype-specific variation was observed for GA composition in root exudates (Fig. 4B). In contrast, hormones profiles measured in root extracts were analyzed independently and are presented in Fig. S1. PC1 was associated with high loading of indole-3-acetic acid (IAA) and tryptamine (TRA), which were most abundant in Baldo (mean IAA: 33.2 pmol/l; TRA: 2.3 pmol/l). In contrast, the inactive conjugated form IAA-glutamate (IAA-Glu) was detectable only in ‘Vialone Nano’ (mean: 4.0 pmol/l). PC2 reflected opposing gradients of indole-3-pyruvic acid (IPyA), which showed the highest abundance in ‘Vialone Nano’ (391.9 pmol/l vs. 179.1 pmol/l in ‘Baldo’ and 158.2 pmol/l in Rufipogon), and 2-oxoindole-3-acetic acid (oxIAA), which increased from ‘Baldo’ (24.4 pmol/l) through Rufipogon (41.8 pmol/l) to ‘Vialone Nano’ (61.4 pmol/l). Overall, genotype accounted for 39% of the variation in the multivariate distance matrix (PERMANOVA: *R*^2^ = 0.39, *F* = 1.96, *p* = 0.034). For gibberellins, concentrations spanned several orders of magnitude, ranging from 0.008 pmol/l (the inactive form GA_34_ in *O. sativa* cv. Baldo) to 956 pmol/l (the inactive form GA_9_ in *O. sativa* cv. Baldo), indicating a highly heterogeneous hormonal profile. PC1 explained 77.1% of the total variance (Fig. 4B) and was primarily associated with the precursor GA_9_ (highest in Baldo, mean: 956.4 pmol/l) and the inactive form GA_51_ (highest in Rufipogon, mean: 577.4 pmol/l), while ‘Vialone Nano’ displayed intermediate values for both compounds (GA_9_: 219.2; GA_51_: 74.8 pmol/l). Among active gibberellins (GA_1_, GA_3_, GA_4_, and GA_7_), GA_4_ was the most abundant, with the highest values observed in ‘Vialone Nano’ (mean: 2.82 pmol/l). All remaining gibberellins, including both precursors and inactive forms, were present at < 5 pmol/l. Genotype explained 53% of the variation in the multivariate distant matrix (PERMANOVA: *R*^2^ = 0.53, *F* = 3.36, *p* = 0.006). Overall, these results indicate distinct genotype-specific phytohormone profiles in root exudates, as reflected by higher explained variance in PERMANOVA.

### Different host genotypes showed diverse transcriptional responses to the same endophytes

RNA-seq analysis of inoculated rice genotypes (Dataset S3) showed that plants were also able to discriminate between bacterial strains. Gene entries collected from the 54 rice plant samples were filtered to retain only genes expressed in at least 3 samples, thereby ensuring consistency across sample replicates. Considering all three plant genotypes (“Overall”), RCA24 affected the expression of a greater number of genes than RCA25 (Table 3 and Table S5). When the three plant genotypes were analyzed separately, *O. rufipogon* (“All”) exhibited the fewest number of DEGs vs the control. In contrast, stems of the ‘Baldo’ genotype showed a markedly stronger transcriptional response to bacterial inoculation, with 3813 DEGs identified following inoculation with RCA24 and RCA25, substantially exceeding the number observed for ‘Vialone Nano’ and *O. rufipogon*. These results indicate that the three plant genotypes differ in their ability to perceive and respond to endophytic bacterial colonization. *O. rufipogon* roots showed the greatest transcriptional response to RCA25 inoculation (Table 3 and Table S5), consistent with a stronger activation of this strain by *O. rufipogon* root exudates (Table 2).

**Table 3:** Number of DEGs identified in *O. sativa* and *O. rufipogon*

	Contrast	N. of DEGs	Site
Overall	RCA24 vs Control	159	All
	RCA25 vs Control	74	All
	RCA25 vs RCA24	93	All
*O. sativa* Baldo	RCA25 vs RCA24	117	All
	RCA25 vs RCA24	42	Root
	RCA25 vs RCA24	3,813	Stem
*O. sativa* Vialone Nano	RCA25 vs RCA24	79	All
	RCA25 vs RCA24	102	Root
	RCA25 vs RCA24	77	Stem
*O. rufipogon*	RCA25 vs RCA24	39	All
	RCA25 vs RCA24	347	Root
	RCA25 vs RCA24	61	Stem

Comparisons with control (blank) and between RCA25 and RCA24 are reported for the whole dataset (All), and the root and stem tissues

**Fig. 5:**
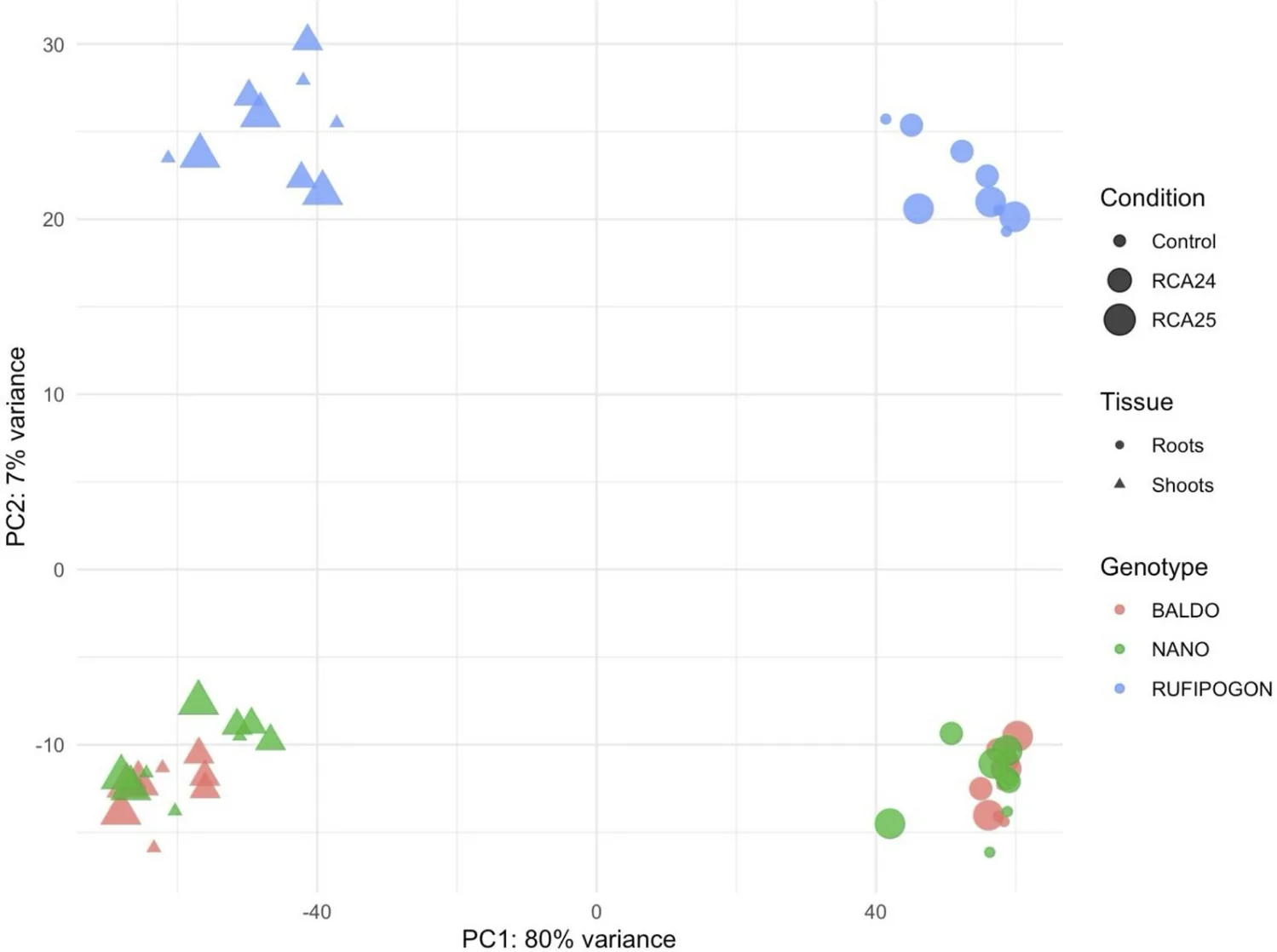
Principal component analysis of rice transcriptome profiles. Each point corresponds to one biological replicate (*n* = 3 per treatment/genotype). PCA was performed using normalized gene expression values. The percentages reported on the axes indicate the proportion of total variance explained by the first two principal components

**Fig. 6:**
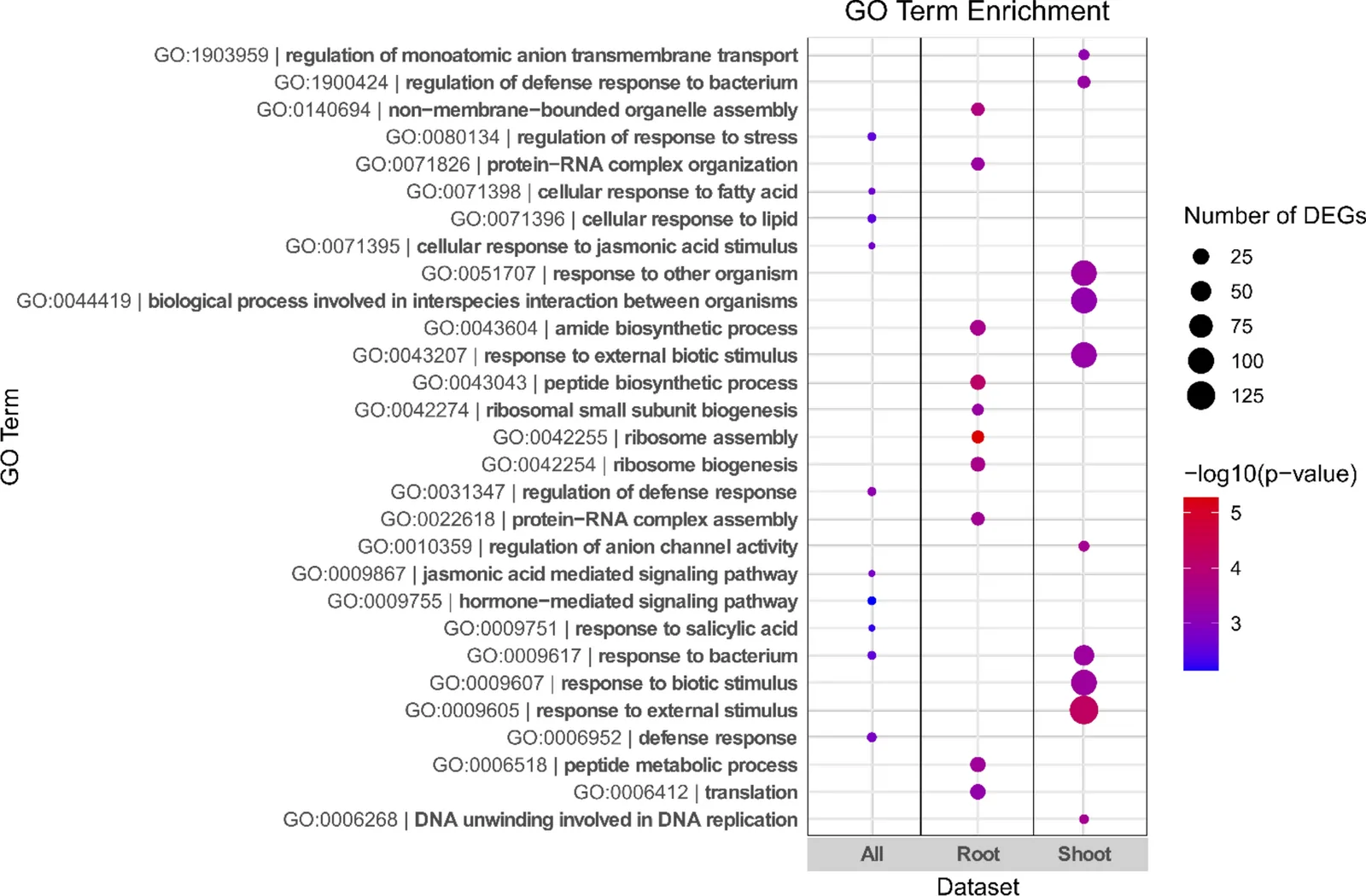
Gene Ontology (GO) enrichment analysis of rice DEGs significantly explained by the interaction term of the nested model. Significance was assessed using topGO (Alexa and Rahnenfuhrer 2024), as described in the Materials and methods. The x-axis shows the three datasets analyzed, whereas the y-axis reports enriched biological process GO terms. Bubble size represents the number of DEGs associated with each GO term, and bubble color indicates enrichment significance (adjusted *p* value). For each dataset, the 10 most significantly enriched GO terms are shown

**Fig. 7:**
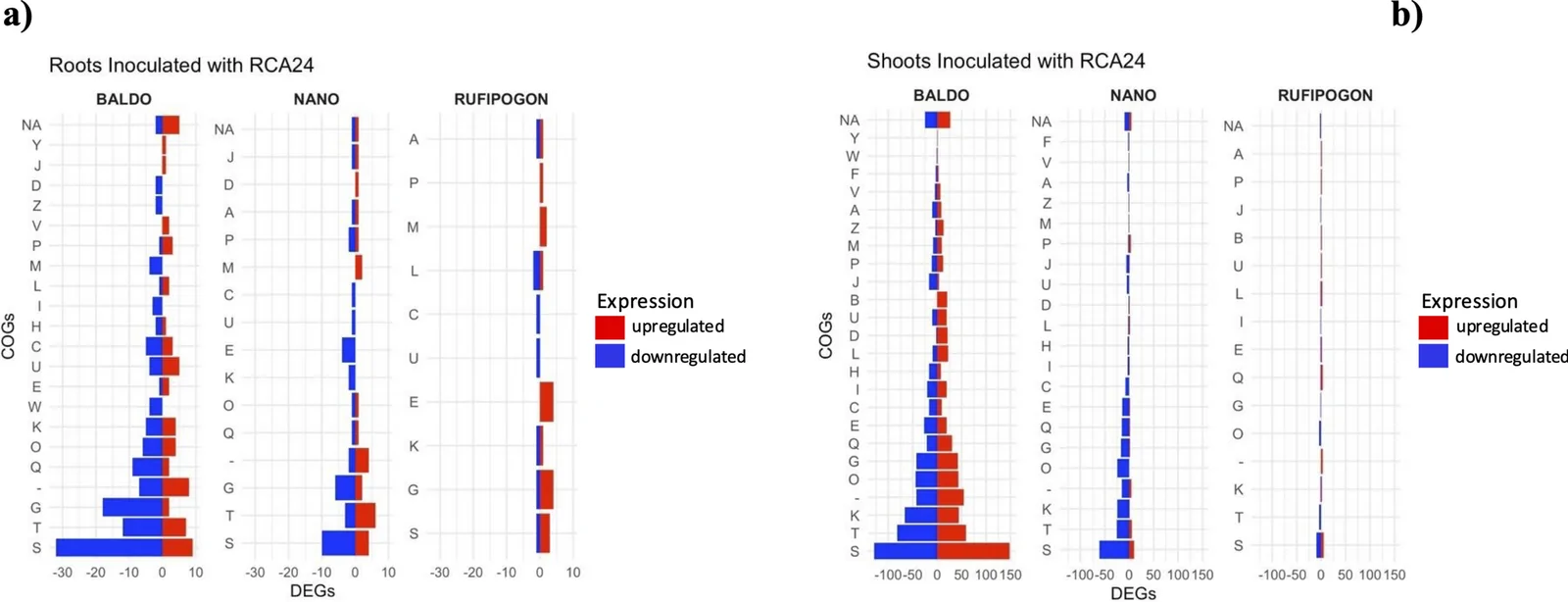
Functional classification of differentially expressed genes (DEGs) in rice identified in response to RCA24 treatment. **A** Roots; **B** shoots. DEGs were identified by comparing each treatment with the corresponding control using DESeq2 package and applying a significance threshold of adjusted p value (FDR) < 0.05 and |log2FC|≥ 1. Functional categories were assigned according to the Clusters of Orthologous Groups (COG) database. Bars represent the percentage of DEGs assigned to each functional category relative to the total number of annotated DEGs in the corresponding dataset. Transcriptome analyses were performed using *n* = 3 biological replicates per treatment. COG category descriptions are reported in Table S1

**Fig. 8:**
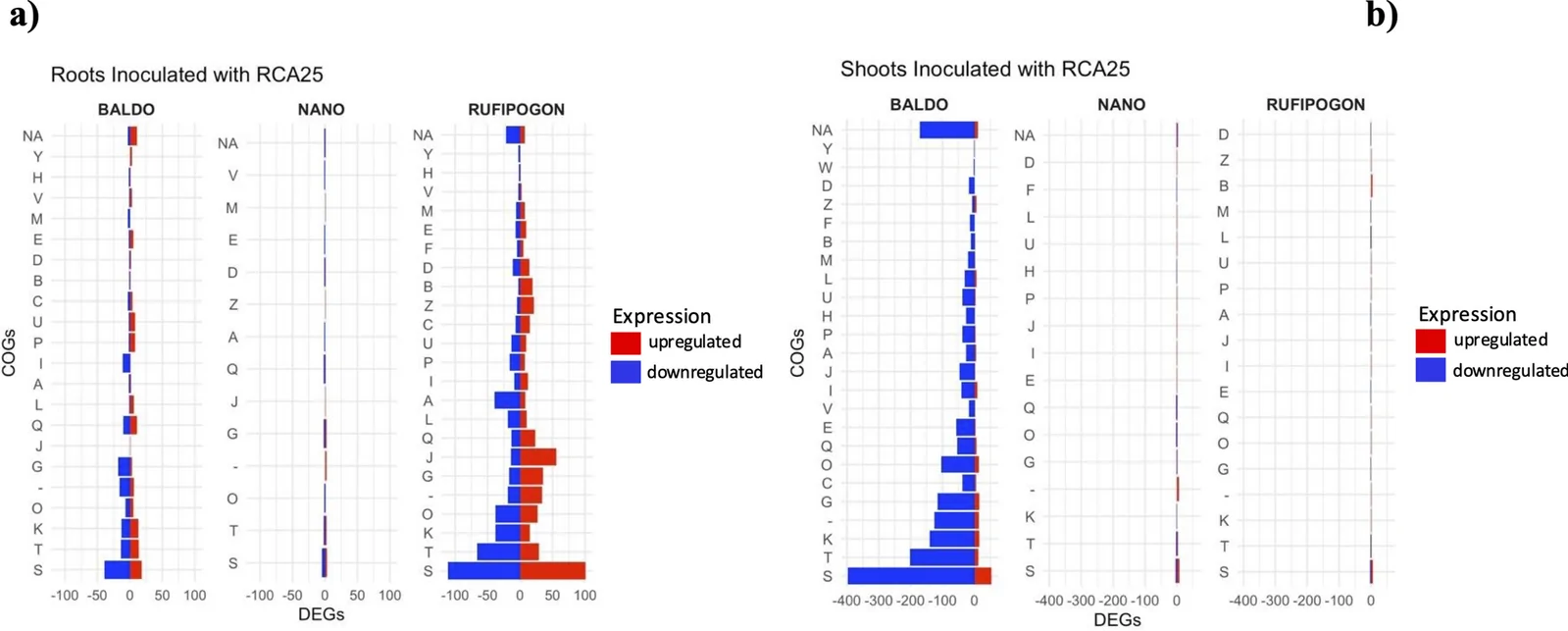
Functional classification of differentially expressed genes (DEGs) in rice identified in response to RCA25 treatment. **A** Roots; **B** shoots. DEGs were identified by comparing each treatment with the corresponding control using DESeq2 package and applying a significance threshold of adjusted *p* value (FDR) < 0.05 and |log2FC|≥ 1. Functional categories were assigned according to the Clusters of Orthologous Groups (COG) database. Bars represent the percentage of DEGs assigned to each functional category relative to the total number of annotated DEGs in the corresponding dataset. Transcriptome analyses were performed using *n* = 3 biological replicates per treatment. COG category descriptions are reported in Table S1

The principal component analysis reported in Fig. 5 showed distinct transcriptional profiles according to both tissue types and plant genotype. A clear separation along the first principal component was observed between roots and shoots, whereas the second principal component separated *O. rufipogon* from *O. sativa* genotype. To further investigate tissue-specific effects, a nested likelihood ratio test (LRT) was also applied to the complete dataset as well as to root- and shoot-specific datasets. Models of increasing complexity were compared to evaluate the contribution of bacterial strain, plant genotype, and their interaction. Among genes whose expression was significantly influenced, plant genotype emerged as the primary source of transcriptional variation, accounting for 5.78% of genes in the complete dataset (Table 4). This effect was even more evident in roots (9.80%) and shoots (6.39%). In contrast, bacterial strain affected a considerably smaller proportion of genes (0.10% overall, 0.05% in roots, and 1.46% in shoots), suggesting a more limited transcriptional effect of strain identity. A small but biologically relevant subset of genes exhibited a significant strain × genotype interaction (0.01% in the full dataset, 0.63% in roots, and 1.55% in shoots), suggesting that specific combinations of rice genotypes and bacterial strains produced distinct transcriptional outcomes. The number of DEGs associated with the interaction was higher when root (764 DEGs, 0.63% of total number of tested genes) and shoot (1883 DEGs, 1.55%) dataset were analyzed separately. Functional enrichment analysis (Fig. 6) of DEGs whose variance was significantly explained by the interaction term showed that the ten most enriched GO terms were primarily associated with plant defense and stress responses, particularly hormone-mediated signaling pathways involved in plant immunity. Terms, such as regulation of defensive response, defensive response, and jasmonic acid-mediated signaling pathway, were significantly overrepresented. In roots, enriched GO terms were mainly associated with fundamental cellular processes related to protein synthesis and ribosome function, suggesting enhanced metabolic activity and growth-related processes. In contrast, shoot-specific DEGs were enriched in GO categories related to responses to external stimuli, response to biotic stimuli, response to bacteria, and regulatory processes, indicating an active role of shoot in signaling and defense responses to environmental and microbial challenges. Comparing the COG categories associated with DEGs in *O. rufipogon* and *O. sativa* inoculated with RCA25 and RCA24 revealed distinct patterns (Fig. 7, Fig. 8, Table S1). In ‘Vialone Nano’ and *O. rufipogon*, most DEGs were upregulated in RCA25 vs. RCA24, whereas in ‘Baldo’ a substantial number of downregulated genes was also observed. Among the assigned functions, signal transduction mechanisms (COG T) and carbohydrate transport and metabolism (COG G) represented the second most abundant categories. However, the largest proportion of DEGs across all three genotypes belongs to COG category S (function unknown), highlighting the limited understanding of the molecular mechanisms underlying plant recognition of endophytic bacteria. This functional classification (Fig. 7, Fig. 8, Table S1) also revealed downregulation of several functional categories, including categories partially associated with defense responses, particularly in shoot tissues. RCA25 stimulated multiple GO categories in *O. rufipogon* roots, whereas the opposite trend was observed in both shoot and root of ‘Baldo’ and ‘Vialone Nano’ (Fig. 7, Fig. 8, Table S1).

**Table 4:** Relevance of plant transcriptome in bacterial strain recognition

		Number of DEGs^a^	
Strain	a) All	b) Root	c) Shoot
Rice genotype	127	61	1793
Strain and rice genotype	7003	11,859	7750
Strain x rice genotype	7130	11,920	9543
None	12	766	1865
Total	84498	69135	77409
Strain	105660 (100%)	105660 (100%)	105660 (100%)

Summary of the number and percentage of significant gene combinations out of the total 121,464 (40,488 genes) identified through a nested likelihood ratio test (LRT)

^**a**^Significance was determined using an FDR-adjusted p-value threshold of 0.05. Models were applied to the a) full transcriptome dataset as well as to b) root and c) shoot subsets. Genes were categorized based on the microbial strain (i.e., differentially expressed only between strains), by the rice genotype (i.e., differentially expressed only between rice genotypes), with both factors independently (i.e., differentially expressed in relation to both rice genotype and microbial strain, but not explained by their interaction), or with the interaction between rice genotype and microbial strain (i.e., expression significantly influenced by the specific combination of genotype and strain)

## Discussion

Understanding the molecular mechanisms underlying the interaction between plants and their associated microbial communities is crucial for the development of sustainable and efficient agricultural practices (Ke et al. 2021; Busby et al. 2017).

Throughout this study, the number of DEGs was interpreted as an indicator of the extent of transcriptional reprogramming induced by plant–bacterium interactions. However, DEG abundance alone does not provide evidence of functional benefits, colonization success, or growth promotion, which require direct phenotypic validation.

Here, we provide molecular insights into rice–endophyte interactions associated with reported differences in bacterial colonization among rice genotypes. The observation that distinct rice genotypes produce different phytohormone cocktails in their root exudates and, consequently, elicit variable transcriptional responses in bacterial strains highlights the importance of genotype-by-genotype interactions in shaping plant–microbe associations. In ‘Vialone Nano’ addition, the observed association between exudate phytohormone composition and bacterial transcriptional responses suggests a potential role for phytohormone-mediated signaling during the early stages of plant–bacterium interactions.

Our results are consistent with previous findings in rhizobium-legume systems, where plant genotype-dependent variation in root exudate composition has been shown to influence the expression of microbial genes crucial for symbiosis formation (Fagorzi et al. 2021; Kiers et al. 2007). Analysis of bacterial transcriptomes following exposure to root exudates revealed distinct responses in RCA24 and RCA25, indicating that bacterial genotype is a major determinant of the interaction. Although these responses may be associated with the different colonization levels previously observed for the two strains, the magnitude of transcriptional reprogramming should not be interpreted as a direct measure of colonization success or functional benefit. For example, RCA25 exhibited a stronger response to root exudates from *O. rufipogon* than those from *O. sativa* accessions, indicating a stronger transcriptional responsiveness of RCA25 to exudates from this ancestral rice genotype. In contrast, RCA24 showed a marked ability to discriminate among exudates derived from different rice genotypes. *O. sativa* variety elicited the highest proportion of unique DEGs, thus indicating that RCA24 can distinguish among exudate profiles produced by different rice genotypes and adjust its gene expression accordingly. Future metabolomic analyses and fractionations of root exudate metabolomes from different rice varieties may help identify potential metabolites responsible for the marked differences observed in the transcriptomic response of RCA24. It is tempting to hypothesize that the differential transcriptional responses observed in the early stages of plant–microbe interactions are associated with later colonization dynamics and plant responses. However, this hypothesis requires direct experimental validation through phenotypic measurements and colonization assays. Since bacterial transcriptomes, plant transcriptomes, and colonization levels were assessed under different experimental conditions and at different time points, and because bacterial responses were measured in vitro following exposure to root exudates, the observed transcriptional patterns should be interpreted as molecular signatures of the interaction rather than direct predictors of colonization outcomes under natural conditions.

Our findings highlighted that *O. rufipogon* root exudates possess distinct chemical signatures that strongly modulate bacterial behavior, suggesting that domestication may have inadvertently altered the root exudates composition and, consequently, the recruitment and activity of specific microbial taxa. Analysis of phytohormone composition of root exudates from *O. rufipogon* and *O. sativa* varieties revealed marked differences in the gibberellin GA_9_ and GA_51_. The biological significance of these differences with respect to transcriptomic response of RCA25, which responded preferentially to *O. rufipogon*, and RCA24, which responded more specifically to *O. sativa* varieties, is beyond the aim of the present study. Nevertheless, it is noteworthy that bacteria possess a cytochrome P450-based pathway for gibberellin biosynthesis (Salazar-Cerezo et al. 2018) and that gibberellin production has been reported in the rice pathogen *Xanthomonas oryzae* pv. *oryzicola* (Nagel et al. 2017). Additionally, IAA was detected at higher concentrations in *O. sativa* varieties than in *O. rufipogon*. Interestingly, substantial IAA production has previously been reported only by RCA24 (Andreozzi et al. 2019). Bacteria are known to perceive IAA, which can regulate biofilm formation, metabolism, auxin catabolism, virulence, chemotactic responses, and host plant colonization (Etesami and Glick 2024; Rico-Jiménez et al. 2024). Moreover, it has been shown that IAA produced by endophytic bacteria play a central role in establishing beneficial plant–microbe interactions by modulating plant physiology. At the same time, pathogenic bacteria can exploit IAA to suppress or overcome plant defense responses (Arora et al. 2024; Feng et al. 2024). In *Serratia plymuthica* A153, like what was observed here for RCA25, IAA induces strong up-regulation of genes involved in cell wall/membrane/envelope biogenesis (COG M), as well as amino acid transport and metabolism (COG E) (Rico-Jiménez et al. 2024). Therefore, we cannot exclude the possibility that the transcriptomic response of *K. sacchari* RCA25 and *E. asburiae* RCA24 were influenced, at least in part, by the differences in phytohormone composition of root exudates. These observations are consistent with previous reports on other grass species (e.g., barley), where domestication has been shown to alter the composition and complexity of root exudates, thereby affecting interactions with beneficial microbes (Escudero-Martinez and Bulgarelli 2019). Examination of the functional categories represented among DEGs, further revealed that perception of root exudates induced extensive remodeling of cellular energy metabolism. Similar remodeling of central metabolic pathways has also been reported in the legume symbiont *S. meliloti* (DiCenzo et al. 2016; Fagorzi et al. 2021). Plant genes involved in shaping microbiota composition have recently been identified in barley, tomato, and sorghum (Wu et al. 2012; Deng et al. 2021; Escudero-Martinez et al. 2022; Oyserman et al. 2022). In rice, it was observed that soil microbiome triggers the downregulation of genes coding for proteins containing NB-ARC domains, including leucine-rich repeat receptor-like kinases and nucleotide-binding leucine-rich repeat receptors (Santos-Medellin et al. 2022). We therefore hypothesize that defense-related pathways may contribute to the transcriptomic differences observed between *O. rufipogon* and *O. sativa*. To test this hypothesis, we performed transcriptomic analyses of *O. rufipogon, O. sativa* ‘Baldo’, and ‘Vialone Nano’ plants colonized by RCA25 or RCA24. Differences in DEGs abundance revealed that RCA24 preferentially modulated gene expression in ‘Vialone Nano’, whereas RCA25 exerted stronger effects in *O. rufipogon* and ‘Baldo’. These patterns were reflected changes in genes known to be involved in endophytic colonization.

Rice transcriptome results revealed that while plant genotype was the primary factor shaping transcriptional responses to bacterial colonization, although strain × genotype interactions affected a biologically significant subset of genes. The hypothesis that plant genotype-specific transcriptomic signatures are associated with bacterial colonization patterns was supported by the differential transcriptional responses observed in *O. rufipogon* colonized by RCA25 and ‘Baldo’ colonized by RCA24. Furthermore, the nested-LRT model showed that the shoot transcriptome displayed stronger interaction effects than the root transcriptome, suggesting that systemic signaling and aboveground perception of bacterial presence may play a key role in determining colonization outcomes. Based on these observations, we hypothesize that the rice response to bacterial colonization becomes more pronounced once bacteria reached the stem tissue, where differences in colonization titers were previously observed. The genes that most clearly differentiated responses to RCA24 and RCA25 were associated with plant defense (e.g., cystatin genes) and hormone-mediated signaling, suggesting that different endophytic strains trigger distinct defense- and hormone-related transcriptional programs in rice plants. These findings are consistent with observations in barley and tomato (Escudero-Martinez et al. 2022; Oyserman et al. 2022), where plant defense regulators such as NPR1 have been shown to underlie microbiota assembly, indicating that conserved defense-related genetic modules may contribute to microbial recruitment across species. Interestingly, both the magnitude and specificity of the plant response differed among rice genotypes. The cultivated variety Baldo displayed a particularly high number of DEGs in stems when comparing RCA24 and RCA25 colonization, whereas the wild ancestor *O. rufipogon* exhibited fewer transcriptional changes in this tissue. This result suggests that domestication may have altered the transcriptional plasticity of rice in response to endophytic colonization, potentially making the two modern cultivars analyzed transcriptionally more responsive to bacterial colonization. Whether these differences translate into altered colonization efficiency or plant performance remains to be determined. Conversely, a significant number of DEGs were observed in roots colonized by RCA25, suggesting the activation of genotype-specific recognition and signaling pathways during this interaction. However, a large proportion of DEGs lacked functional annotation, indicating that many of the genetic determinants underlying plant–microbe interactions remain unknown in both plant and bacterial genomes.

The experimental system used in this study was necessarily simplified, involving three rice genotypes and two bacterial strains isolated from the same cultivated rice field. Natural plant–microbiota interactions are considerably more complex and involve simultaneous interactions among plants, diverse microbial communities. Nonetheless, our results revealed substantial variability in the response of both plants and bacteria, as well as among different plant compartments.

Overall, the results presented here underscore the remarkable complexity of plant–microbiota interactions, a complexity that has likely been underestimated. Further studies integrating metabolomic profiling with plant and bacterial transcriptomes will help determine whether hormone composition directly drives transcriptional changes in plants and bacteria or whether these effects are mediated indirectly through microbial metabolism.

Given that crop plants have adapted to agricultural environments that may have reduced some of the traits retained by their wild relatives, our findings provide a framework for investigating how plant genetic diversity and microbial diversity shape molecular interactions between plants and endophytes. Further studies linking transcriptomic responses to direct measurements of colonization efficiency and plant performance will be necessary to determine the functional significance of these molecular signatures (Raaijmakers and Kiers 2022).

## Conclusions

This study demonstrates that wild and cultivated rice accessions exhibit distinct transcriptional programs both in shaping bacterial responses to root exudates and in responding to endophytic colonization. The two bacterial strains RCA24 and RCA25 displayed divergent transcriptional strategies: RCA24 differentiated between the two *O. sativa* varieties, while RCA25 showed more extensive responses to *O. rufipogon* exudates, suggesting that ancestral rice maintains a richer molecular dialog with beneficial bacteria. At the plant level, colonization by RCA24 induced broader transcriptional reprogramming than RCA25, particularly in shoots, involving defense responses, hormone signaling, and ribosome biogenesis, highlighting genotype-dependent responses to different bacterial strains.

Collectively, these findings suggest that traits governing plant–microbiota interactions in *O. rufipogon* have been partially lost during domestication and may represent valuable targets for improving microbial association in cultivated rice. Future studies integrating metabolomics with plant and bacterial transcriptomes will be critical to disentangle whether hormonal signals in root exudates directly trigger bacterial gene expression or whether plant responses are indirectly shaped by bacterial metabolism. A deeper understanding of the genetic basis of these interactions could ultimately inform breeding programs aimed at developing rice cultivars with enhanced beneficial microbial associations and improved agronomic performance.

## Supplementary Information

Below is the link to the electronic supplementary material.

Supplementary file1 (XLSX 21 KB)

Supplementary file2 (XLSX 26 KB)

Supplementary file3 (DOCX 10133 KB)

Supplementary file4 (TSV 78 KB)

Supplementary file5 (TSV 58 KB)

Supplementary file6 (TSV 78 KB)

Supplementary file7 (TSV 10 KB)

Supplementary file8 (TSV 30 KB)

Supplementary file9 (DOCX 16 KB)

## Data Availability

Sequence reads of RNA-seq have been deposited and are available as Projects PRJEB82954 (ENA database, bacteria RNA-Seq data) and PRJNA1309287 (NCBI database, plant RNA-Seq data). All the custom code used for preprocessing and data analysis is available at the following GitHub link: https://github.com/IacopoPasseri/RIS8-Ricebiota.
