# Supplementary material for: Comparative transcriptomic analysis reveals distinct responses of beneficial bacterial endophytes to wild and cultivated rice root exudates
Source: Plant Cell Rep. 2026 Aug 20;45(9):252. doi: 10.1007/s00299-026-03936-0 (PMC13493465; doi:10.1007/s00299-026-03936-0)
Supplement: Supplementary file 1 [file 299_2026_3936_MOESM3_ESM.docx]

**Supplementary Tables and Figures**

**Table S1**. Description of single-letter COG codes.

INFORMATION STORAGE AND PROCESSING

[J] Translation. ribosomal structure and biogenesis

[A] RNA processing and modification

[K] Transcription

[L] Replication. recombination and repair

[B] Chromatin structure and dynamics

CELLULAR PROCESSES AND SIGNALING

[D] Cell cycle control. cell division. chromosome partitioning

[Y] Nuclear structure

[V] Defense mechanisms

[T] Signal transduction mechanisms

[M] Cell wall/membrane/envelope biogenesis

[N] Cell motility

[Z] Cytoskeleton

[W] Extracellular structures

[U] Intracellular trafficking. secretion. and vesicular transport

[O] Posttranslational modification. protein turnover. chaperones

METABOLISM

[C] Energy production and conversion

[G] Carbohydrate transport and metabolism

[E] Amino acid transport and metabolism

[F] Nucleotide transport and metabolism

[H] Coenzyme transport and metabolism

[I] Lipid transport and metabolism

[P] Inorganic ion transport and metabolism

[Q] Secondary metabolites biosynthesis. transport and catabolism

POORLY CHARACTERIZED

[R] General function prediction only

[S] Function unknown

**Table S2**. Mean concentrations (pmol/l ± SD. n = 3) of *auxins* in root exudates and extracts of O. sativa cv. Baldo. O. rufipogon. and O. sativa cv. Vialone Nano. Values below the limit of detection are reported as the LOD threshold and highlighted in bold.

| **Compound** |  | **Baldo** | **Rufipogon** | **V. Nano** |
| --- | --- | --- | --- | --- |
| **ANT** | **exudate** | 45.4333 ± 49.7968 | 16.3667 ± 8.3339 | 26.0000 ± 13.6963 |
|  | **root extract** | 495.9333 ± 91.1114 | 875.5333 ± 334.3919 | 2243.0000 ± 1245.7953 |
| **IAA** | **exudate** | 33.2000 ± 30.6886 | 20.2667 ± 7.4849 | 10.7000 ± 4.6893 |
|  | **root extract** | 1177.8667 ± 73.8419 | 411.0333 ± 76.4690 | 4840.9333 ± 3103.4373 |
| **IAA-Asp** | **exudate** | 0.4767 ± 0.4210 | 0.3202 ± 0.2770 | 0.8367 ± 0.3609 |
|  | **root extract** | 678.6233 ± 98.9018 | 125.0133 ± 124.1091 | 1506.3567 ± 639.0733 |
| **IAA-Glu** | **exudate** | **0.0050 ± 0.0000** | **0.0050 ± 0.0000** | 4.0000 ± 0.2646 |
|  | **root extract** | 425.6333 ± 88.1104 | 35.2000 ± 10.0583 | 842.3333 ± 67.7521 |
| **IAA-glc** | **exudate** | **0.0050 ± 0.0000** | **0.0050 ± 0.0000** | **0.0050 ± 0.0000** |
|  | **root extract** | **0.0050 ± 0.0000** | 24.3667 ± 7.9103 | 15.3683 ± 14.6576 |
| **IAM** | **exudate** | 0.6767 ± 0.6199 | 0.2633 ± 0.1686 | 0.8800 ± 0.4838 |
|  | **root extract** | 26.0700 ± 6.5608 | 15.6800 ± 3.0086 | 28.6333 ± 5.9057 |
| **IAN** | **exudate** | **0.0100 ± 0.0000** | **0.0100 ± 0.0000** | **0.0100 ± 0.0000** |
|  | **root extract** | **0.0100 ± 0.0000** | **0.0100 ± 0.0000** | **0.0100 ± 0.0000** |
| **IPyA** | **exudate** | 179.1667 ± 89.8255 | 158.2000 ± 103.3561 | 392.0000 ± 154.3210 |
|  | **root extract** | 30795.0000 ± 1480.8507 | 13627.9000 ± 5074.4723 | 49191.8000 ± 21702.7721 |
| **TRA** | **exudate** | 2.3233 ± 2.1495 | 0.8200 ± 0.0265 | 0.8333 ± 0.8702 |
|  | **root extract** | 381.3667 ± 124.1259 | 298.4367 ± 74.9507 | 425.3967 ± 174.6320 |
| **TRP** | **exudate** | **0.0100 ± 0.0000** | **0.0100 ± 0.0000** | **0.0100 ± 0.0000** |
|  | **root extract** | 1176752.2667 ± 175454.2987 | 1193205.7000 ± 202279.8735 | 1656015.3333 ± 575500.8945 |
| **oxIAA** | **exudate** | 24.4000 ± 23.7956 | 41.8000 ± 35.0801 | 61.4333 ± 18.0059 |
|  | **root extract** | 206.5667 ± 72.0723 | 72.7333 ± 16.9223 | 651.0333 ± 123.2742 |
| **oxIAA-Asp** | **exudate** | 0.1137 ± 0.1470 | 0.5567 ± 0.4535 | 0.4633 ± 0.2650 |
|  | **root extract** | 88.4500 ± 8.4143 | 79.4967 ± 62.8399 | 376.6667 ± 288.4398 |
| **oxIAA-Glu** | **exudate** | **0.0050 ± 0.0000** | **0.0050 ± 0.0000** | **0.0050 ± 0.0000** |
|  | **root extract** | **0.0005 ± 0.0000** | **0.0005 ± 0.0000** | 13.0333 ± 5.8535 |
| **oxIAA-glc** | **exudate** | **0.0050 ± 0.0000** | **0.0050 ± 0.0000** | **0.0050 ± 0.0000** |
|  | **root extract** | 83.3000 ± 15.2148 | 27.0000 ± 11.9528 | 83.2000 ± 13.0817 |

**Table S3**. Mean concentrations (pmol/l ± SD. n = 3) of *gibberellins* in root exudates and extracts of O. sativa cv. Baldo. O. rufipogon. and O. sativa cv. Vialone Nano. Values below the limit of detection are reported as the LOD threshold and highlighted in bold.

| **Compound** |  | **Baldo** | **Rufipogon** | **V. Nano** |
| --- | --- | --- | --- | --- |
| **GA1** | **exudate** | 0.4167 ± 0.0404 | 0.4233 ± 0.0321 | 0.8233 ± 0.2511 |
|  | **root extract** | 10.3767 ± 4.0501 | 4.1567 ± 0.2301 | 4.8467 ± 0.9136 |
| **GA15** | **exudate** | 0.0043 ± 0.0000 | 0.0043 ± 0.0000 | 0.0043 ± 0.0000 |
|  | **root extract** | **0.0043 ± 0.0000** | **0.0043 ± 0.0000** | **0.0043 ± 0.0000** |
| **GA19** | **exudate** | 0.0773 ± 0.0170 | 0.0863 ± 0.0342 | 0.1140 ± 0.0250 |
|  | **root extract** | 1.0660 ± 0.3461 | 0.6597 ± 0.1007 | 0.8350 ± 0.2114 |
| **GA20** | **exudate** | 3.7533 ± 0.8580 | 1.5433 ± 1.0782 | 3.0567 ± 0.1779 |
|  | **root extract** | 1.2900 ± 0.1345 | 1.5833 ± 0.4744 | 1.2067 ± 0.3252 |
| **GA24** | **exudate** | **0.0095 ± 0.0000** | **0.0095 ± 0.0000** | **0.0095 ± 0.0000** |
|  | **root extract** | **0.0095 ± 0.0000** | **0.0095 ± 0.0000** | **0.0095 ± 0.0000** |
| **GA29** | **exudate** | 0.1133 ± 0.0493 | 0.0800 ± 0.0361 | 0.1867 ± 0.0451 |
|  | **root extract** | 1.1733 ± 0.2350 | 0.7700 ± 0.3041 | 0.4633 ± 0.2616 |
| **GA3** | **exudate** | 1.7167 ± 0.0289 | 1.6500 ± 0.0600 | 1.6600 ± 0.0300 |
|  | **root extract** | 7.1533 ± 0.2403 | 6.7567 ± 0.2237 | 7.4000 ± 0.0265 |
| **GA34** | **exudate** | 0.0077 ± 0.0021 | 0.0100 ± 0.0053 | 0.0433 ± 0.0058 |
|  | **root extract** | **0.0058 ± 0.0000** | **0.0058 ± 0.0000** | 0.0913 ± 0.0696 |
| **GA4** | **exudate** | 1.1367 ± 0.5262 | 0.3100 ± 0.1179 | 2.8167 ± 1.1951 |
|  | **root extract** | 3.2300 ± 2.6601 | 1.2567 ± 0.2201 | 6.3700 ± 6.5314 |
| **GA44** | **exudate** | 0.1833 ± 0.1474 | 0.0186 ± 0.0187 | 0.0057 ± 0.0000 |
|  | **root extract** | 1.5800 ± 0.8643 | 1.3467 ± 0.8658 | 0.7352 ± 0.6450 |
| **GA51** | **exudate** | 11.2367 ± 19.4538 | 577.4333 ± 459.0523 | 74.8333 ± 120.7850 |
|  | **root extract** | **0.0051 ± 0.0000** | **0.0051 ± 0.0000** | 23.3000 ± 36.2352 |
| **GA53** | **exudate** | 83.2000 ± 10.6691 | 66.9000 ± 10.8917 | 84.2667 ± 31.0536 |
|  | **root extract** | 95.0000 ± 11.5295 | 95.7333 ± 8.9668 | 122.4000 ± 67.6610 |
| **GA6** | **exudate** | 0.0087 ± 0.0006 | 0.0090 ± 0.0010 | 0.0083 ± 0.0021 |
|  | **root extract** | 0.0213 ± 0.0023 | 0.0230 ± 0.0130 | 0.0171 ± 0.0089 |
| **GA7** | **exudate** | 0.0433 ± 0.0058 | 0.0300 ± 0.0100 | 0.0367 ± 0.0058 |
|  | **root extract** | 0.3500 ± 0.0458 | 0.3700 ± 0.0436 | 0.2800 ± 0.0436 |
| **GA8** | **exudate** | 0.1767 ± 0.0635 | 0.1333 ± 0.0551 | 0.2400 ± 0.0624 |
|  | **root extract** | 0.7300 ± 0.1400 | 0.8300 ± 0.0964 | 0.7133 ± 0.1415 |
| **GA9** | **exudate** | 956.4333 ± 856.1411 | 13.2333 ± 14.1797 | 219.2000 ± 225.8027 |
|  | **root extract** | 2526.5333 ± 2777.7548 | 687.8667 ± 962.6672 | 22947.6667 ± 36851.4079 |

**Table S4**. Description of auxins codes.

| **Compound name** | **Abbreviation** |
| --- | --- |
| Anthranilic acid | ANT |
| Tryptophan | TRP |
| Tryptamine | TRA |
| Indole-3-acetamid | IAM |
| Indole-3-acetonitrile | IAN |
| Indole-3-pyruvic acid | IPyA |
| Indole-3-acetic acid | IAA |
| IAA-aspartate | IAA-Asp |
| IAA-glutamate | IAA-Glu |
| 2-oxoindole-3-acetic acid | oxIAA |
| oxIAA-aspartate | oxIAA-Asp |
| oxIAA-glutamate | oxIAA-Glu |
| IAA-glucose | IAA-glc |
| oxIAA-glucose | oxIAA-glc |

**Table S5**. Number of DEGs identified in *O. sativa* and *O. rufipogon* for all analyzed contrasts.

|  | **Contrast** | **n. of DEGs** | **Site** |
| --- | --- | --- | --- |
| Overall | RCA24 vs Control | 159 | All |
|  | RCA25 vs Control | 74 | All |
|  | RCA25 vs RCA24 | 93 | All |
| *O. sativa* “Baldo” | RCA24 vs Control | 165 | All |
|  | RCA25 vs Control | 172 | All |
|  | RCA25 vs RCA24 | 117 | All |
|  | RCA24 vs Control | 168 | Root |
|  | RCA25 vs Control | 274 | Root |
|  | RCA25 vs RCA24 | 42 | Root |
|  | RCA24 vs Control | 1202 | Stem |
|  | RCA25 vs Control | 1870 | Stem |
|  | RCA25 vs RCA24 | 3.813 | Stem |
| *O. sativa* “Vialone Nano” | RCA24 vs Control | 92 | All |
|  | RCA25 vs Control | 16 | All |
|  | RCA25 vs RCA24 | 79 | All |
|  | RCA24 vs Control | 51 | Root |
|  | RCA25 vs Control | 31 | Root |
|  | RCA25 vs RCA24 | 102 | Root |
|  | RCA24 vs Control | 274 | Stem |
|  | RCA25 vs Control | 45 | Stem |
|  | RCA25 vs RCA24 | 77 | Stem |
| *O. rufipogon* | RCA24 vs Control | 35 | All |
|  | RCA25 vs Control | 38 | All |
|  | RCA25 vs RCA24 | 39 | All |
|  | RCA24 vs Control | 24 | Root |
|  | RCA25 vs Control | 903 | Root |
|  | RCA25 vs RCA24 | 347 | Root |
|  | RCA24 vs Control | 46 | Stem |
|  | RCA25 vs Control | 33 | Stem |
|  | RCA25 vs RCA24 | 61 | Stem |

**Table S6.** Gene sets (KEGG pathways and COG categories) significantly enriched (adjusted p-value < 0.05) by cutoff-free GSEA for each contrast and bacterial strain. using the DESeq2 Wald statistic as the ranking metric for the Baldo vs control contrast. Full results for all remaining contrasts and bacterial strains are provided in the Zenodo repository (doi:10.5281/zenodo.21397747).

| **Strain** | **Contrast** | **pathway** | **pval** | **padj** | **log2err** | **ES** | **NES** | **Size** | **Pathway name** |
| --- | --- | --- | --- | --- | --- | --- | --- | --- | --- |
| RCA24 | Baldo vs control | map00190 | 1.146e-07 | 1.456e-05 | 0.7050 | 0.64317 | 2.39725 | 44 | Oxidative phosphorylation |
| RCA24 | Baldo vs control | map03010 | 3.265e-07 | 2.073e-05 | 0.6750 | 0.59573 | 2.27872 | 52 | Ribosome |
| RCA24 | Baldo vs control | map00270 | 3.413e-05 | 0.0014 | 0.5573 | 0.56409 | 2.12000 | 46 | Cysteine and methionine metabolism |
| RCA24 | Baldo vs control | map00010 | 0.0002 | 0.0078 | 0.4985 | 0.47283 | 1.87742 | 62 | Glycolysis / Gluconeogenesis |
| RCA24 | Baldo vs control | map02040 | 0.0005 | 0.0130 | 0.4773 | 0.54608 | 1.96603 | 36 | Flagellar assembly |
| RCA24 | Baldo vs control | map01230 | 0.0007 | 0.0146 | 0.4773 | 0.35773 | 1.64915 | 140 | Biosynthesis of amino acids |
| RCA24 | Baldo vs control | map00195 | 0.0010 | 0.0171 | 0.4551 | 0.84565 | 1.89480 | 7 | Photosynthesis |
| RCA24 | Baldo vs control | map00400 | 0.0011 | 0.0171 | 0.4551 | 0.61483 | 1.96622 | 23 | Phenylalanine. tyrosine and tryptophan biosynthesis |
| RCA24 | Baldo vs control | map00730 | 0.0020 | 0.0285 | 0.4317 | -0.64417 | -1.96241 | 17 | Thiamine metabolism |
| RCA25 | Baldo vs control | map01110 | 3.895e-05 | 0.0049 | 0.5573 | 0.30812 | 1.59112 | 328 | Biosynthesis of secondary metabolites |
| RCA25 | Baldo vs control | map00051 | 8.033e-05 | 0.0051 | 0.5384 | 0.56092 | 2.02790 | 38 | Fructose and mannose metabolism |
| RCA25 | Baldo vs control | map00270 | 0.0003 | 0.0083 | 0.4985 | 0.51314 | 1.87768 | 42 | Cysteine and methionine metabolism |
| RCA25 | Baldo vs control | map01230 | 0.0002 | 0.0083 | 0.5188 | 0.36690 | 1.68964 | 131 | Biosynthesis of amino acids |
| RCA25 | Baldo vs control | map02040 | 0.0003 | 0.0087 | 0.4985 | -0.49846 | -1.87273 | 49 | Flagellar assembly |
| RCA25 | Baldo vs control | map00030 | 0.0005 | 0.0113 | 0.4773 | 0.55029 | 1.95611 | 36 | Pentose phosphate pathway |
| RCA25 | Baldo vs control | map01120 | 0.0012 | 0.0205 | 0.4551 | 0.29915 | 1.46856 | 238 | Microbial metabolism in diverse environments |
| RCA25 | Baldo vs control | map01130 | 0.0013 | 0.0205 | 0.4551 | 0.30603 | 1.49527 | 228 | Biosynthesis of antibiotics |
| RCA25 | Baldo vs control | map03010 | 0.0017 | 0.0235 | 0.4551 | -0.46314 | -1.75824 | 51 | Ribosome |
| RCA25 | Baldo vs control | map00250 | 0.0034 | 0.0431 | 0.4317 | 0.50319 | 1.73733 | 31 | Alanine. aspartate and glutamate metabolism |

**Table S6.** *Continued*

| **Strain** | **Contrast** | **COG category** | **pval** | **padj** | **log2err** | **ES** | **NES** | **Size** | **Description** |
| --- | --- | --- | --- | --- | --- | --- | --- | --- | --- |
| RCA24 | Baldo vs control | K | 0.0013 | 0.0227 | 0.4551 | -0.26316 | -1.43625 | 369 | Transcription |
| RCA24 | Baldo vs control | J | 0.0062 | 0.0437 | 0.4070 | 0.30706 | 1.46700 | 187 | Translation. ribosomal structure and biogenesis |
| RCA24 | Baldo vs control | P | 0.0073 | 0.0437 | 0.4070 | 0.27938 | 1.42337 | 294 | Inorganic ion transport and metabolism |
| RCA25 | Baldo vs control | G | 0.0002 | 0.0041 | 0.5188 | 0.31710 | 1.56681 | 223 | Carbohydrate transport and metabolism |
| RCA25 | Baldo vs control | C | 0.0009 | 0.0080 | 0.4773 | 0.29477 | 1.50030 | 277 | Energy production and conversion |
| RCA25 | Baldo vs control | J | 0.0021 | 0.0124 | 0.4317 | -0.32043 | -1.52588 | 183 | Translation. ribosomal structure and biogenesis |
| RCA25 | Baldo vs control | E | 0.0031 | 0.0142 | 0.4317 | 0.27656 | 1.40719 | 280 | Amino acid transport and metabolism |
| RCA25 | Baldo vs control | V | 0.0074 | 0.0268 | 0.4070 | -0.45319 | -1.67909 | 45 | Defense mechanisms |
| RCA25 | Baldo vs control | U | 0.0250 | 0.0751 | 0.3525 | -0.38778 | -1.49237 | 51 | Intracellular trafficking. secretion. and vesicular transport |

**Table S7.** Number of DEGs identified for each contrast and bacterial strain under three log2FC threshold schemes (|log2FC| ≥ 1. |log2FC| ≥ 0.5 and no log2FC cutoff). all with an adjusted p-value < 0.05.

| **Strain** | **Contrast** | **N° total tested** | **DEG LFC1** | **Up**  **LFC1** | **Down LFC1** | **DEG LFC0.5** | **Up LFC0.5** | **Down LFC0.5** | **DEG no FLC cutoff** | **Up no FLC cutoff** | **Down no FLC cutoff** |
| --- | --- | --- | --- | --- | --- | --- | --- | --- | --- | --- | --- |
| **RCA24** | Baldo vs Control | 1886 | 2 | 1 | 1 | 19 | 17 | 2 | 27 | 23 | 4 |
| **RCA24** | Rufipogon vs Control | 3807 | 41 | 31 | 10 | 271 | 140 | 131 | 523 | 247 | 276 |
| **RCA24** | Vialone Nano vs Control | 4225 | 107 | 82 | 25 | 570 | 320 | 250 | 922 | 471 | 451 |
| **RCA24** | Baldo vs Rufipogon | 3557 | 15 | 3 | 12 | 119 | 57 | 62 | 237 | 139 | 98 |
| **RCA24** | Vialone Nano vs Rufipogon | 4225 | 57 | 28 | 29 | 389 | 190 | 199 | 706 | 355 | 351 |
| **RCA25** | Baldo vs Control | 4575 | 1 | 0 | 1 | 5 | 3 | 2 | 6 | 4 | 2 |
| **RCA25** | Rufipogon vs Control | 4486 | 235 | 118 | 117 | 792 | 348 | 444 | 915 | 417 | 498 |
| **RCA25** | Vialone Nano vs Control | 4575 | 15 | 7 | 8 | 38 | 17 | 21 | 42 | 20 | 22 |
| **RCA25** | Baldo vs Rufipogon | 4397 | 70 | 23 | 47 | 225 | 134 | 91 | 259 | 158 | 101 |
| **RCA25** | Vialone Nano vs Rufipogon | 4486 | 63 | 9 | 54 | 160 | 64 | 96 | 171 | 72 | 99 |

**Fig. S1.** Principal Component Analysis biplots of auxins (A) and gibberellins (B) profiles in root extracts of the three genotypes. Dots represent biological replicates. while vectors represent metabolite loadings. The percentage of variance explained by PC1 and PC2 is shown on the axes.
