# Supplementary material for: Comparative transcriptomic analysis reveals distinct responses of beneficial bacterial endophytes to wild and cultivated rice root exudates
Source: Plant Cell Rep. 2026 Aug 20;45(9):252. doi: 10.1007/s00299-026-03936-0 (PMC13493465; doi:10.1007/s00299-026-03936-0)
Supplement: Supplementary file 2 [file 299_2026_3936_MOESM9_ESM.docx]

**Dataset S1.** List of differentially expressed genes identified in *E. asburiae* RCA24 and *K. sacchari* RCA25 following treatment with root exudates from *O. sativa* ('Baldo', 'Vialone Nano') and *O. rufipogon*, compared with untreated control cultures. List of differentially expressed genes identified by comparing transcription profiles of plants colonized with *E. asburiae* RCA24 and *K. sacchari* RCA25 and uninoculated control ones.

**Dataset S2.** Details of metabolic pathways (KEGG mapped) of differentially expressed genes identified for *E. asburiae* RCA24 and *K. sacchari* RCA25 treated with rice root exudates.

**Dataset S3**. List of differentially expressed genes identified by comparing transcription profiles of plants colonized with *E. asburiae* RCA24 and *K. sacchari* RCA25 and uninoculated control ones.
